# Efficacy and safety of tranexamic acid in prevention of postpartum hemorrhage: a systematic review and meta-analysis of 18,649 patients

**DOI:** 10.1186/s12884-023-06100-8

**Published:** 2023-11-24

**Authors:** Nada Mostafa Al-dardery, Omar Ahmed Abdelwahab, Mohamed Abouzid, Khaled Albakri, Ali Elkhadragy, Basant E. Katamesh, Rawan Hamamreh, Ahmed B. Mohd, Ahmed Abdelaziz, Abdulrhman Khaity

**Affiliations:** 1 Faculty of Medicine, Fayoum University, Fayoum, Egypt; 2 Medical Research Group of Egypt (MRGE), Cairo, Egypt; 3 Faculty of Medicine, Al-Azhar University, Cairo, Egypt; 4 Department of Physical Pharmacy and Pharmacokinetics, Poznan University of Medical Sciences, Poznan, Poland; 5 Doctoral School, Poznan University of Medical Sciences, Poznan, Poland; 6 Faculty of Medicine, The Hashemite University, Zarqa, Jordan; 7 Faculty of Medicine, Alexandria University, Alexandria, Egypt; 8 Faculty of Medicine, Tanta University, Tanta, Egypt; 9 Faculty of Medicine, Elrazi University, Khartoum, 11115 Sudan

**Keywords:** Postpartum hemorrhage, Tranexamic acid, Vaginal birth, Cesarean birth

## Abstract

**Background:**

In this meta-analysis, we aimed to update the clinical evidence regarding the efficacy and safety of TXA in the prevention of PPH.

**Methods:**

A literature search of PubMed, Scopus, Web of Science, Google Scholar, and Cochrane Library from inception until December 2022 was conducted. We included randomized controlled trials (RCTs) comparing TXA with a placebo among pregnant women. All relevant outcomes, such as total blood loss, the occurrence of nausea and/or vomiting, and changes in hemoglobin, were combined as odds ratios (OR) or mean differences (MD) in the meta-analysis models using STATA 17 MP.

**Results:**

We included 59 RCTs (18,649 patients) in this meta-analysis. For cesarean birth, TXA was favored over the placebo in reducing total blood loss (MD= -2.11 mL, 95%CI [-3.09 to -1.14], *P* < 0.001), and occurrence of nausea or/and vomiting (OR = 1.36, 95%CI [1.07 to 1.74], *P* = 0.01). For vaginal birth, the prophylactic use of TXA was associated with lower total blood loss, and higher occurrence of nausea and/or vomiting (MD= -0.89 mL, 95%CI [-1.47 to -0.31], OR = 2.36, 95%CI [1.32 to 4.21], *P* = 0.02), respectively. However, there were no differences between the groups in changes in hemoglobin during vaginal birth (MD = 0.20 g/dl, 95%CI [-0.07 to 0.48], *P* = 0.15). The overall risk of bias among the included studies varies from low to high risk of bias using ROB-II tool for RCTs.

**Conclusions:**

This meta-analysis suggested that TXA administration is effective among women undergoing cesarean birth or vaginal birth in lowering total blood loss and limiting the occurrence of PPH. Further clinical trials are recommended to test its efficacy on high-risk populations.

**Supplementary Information:**

The online version contains supplementary material available at 10.1186/s12884-023-06100-8.

## Background

Postpartum hemorrhage (PPH) is a serious complication that can occur after childbirth. It is defined as the loss of more than 500 ml of blood after vaginal birth (VB) or more than 1000 ml after caesarean birth (CB) [1]. PPH is one of the leading causes of maternal mortality worldwide, accounting for approximately one-quarter of all maternal deaths [2].

The most common cause of PPH is loss of uterine tone, trauma during birth throughout, or retained placental tissue. If left untreated, PPH can lead to severe complications such as shock and even death [3–5]. There are several risk factors for PPH, including prolonged labor, multiple pregnancies, previous history of PPH, certain medical conditions such as hypertension and placenta previa, use of forceps or vacuum-assisted birth, and general anesthesia [3, 5].

The management of PPH depends on the severity and underlying cause of the bleeding. It is worth mentioning that prevention is key to reducing the incidence of PPH [1]. Tranexamic acid (TXA) is an antifibrinolytic agent that has been used for many years to reduce bleeding in various surgical procedures [6–12].

TXA, a synthetic derivative derived from lysine, functions by competitively obstructing the binding sites for lysine on plasminogen. Plasminogen possesses five TXA binding sites, with one having a notably strong affinity and the remaining four exhibiting lower affinity [13]. TXA hinders the interaction between plasminogen (the precursor enzyme) and plasmin, consequently inhibiting the activation of plasmin. Furthermore, it obstructs the attachment of plasmin (the active form) to fibrin, leading to the suppression of fibrinolysis. As a result, its primary mechanism of action revolves around stabilizing pre-existing blood clots rather than facilitating new clot formation. It’s essential to underscore that TXA functions as an antifibrinolytic agent, emphasizing its distinction from antihemorrhagic agents [14]. Also, increased concentrations of TXA may have an anti-inflammatory impact by diminishing the proinflammatory effects of plasmin on the complement system [15].

In recent years, TXA has gained attention as an effective treatment for PPH. Several studies have investigated the efficacy and safety of TXA in PPH [7, 11, 12, 16]. The WOMAN trial [17], which was a large randomized controlled trial involving over 20,000 women from 21 countries, found that TXA reduced the risk of death due to bleeding by 31% when given within three hours of birth throughout. Another study conducted in Nigeria also showed that TXA reduced the need for blood transfusion and hysterectomy in women with PPH [18].

While some studies have shown promising results of TXA, limited studies represented a full understanding of its effectiveness and potential side effects [7–9, 11, 12]. Therefore, this meta-analysis aims to update current clinical evidence and determine the clinical efficacy and safety of TXA in the prevention of PPH. We collected up-to-date studies on both CB and VB, which will assist physicians in deciding whether to include TXA in their routine preoperative prophylaxis for PPH.

## Methods

This meta-analysis was conducted in accordance to the Preferred Reporting Items for Systematic Reviews and Meta-Analysis guidelines (PRISMA) [19]. Guidelines of the Cochrane Handbook of Systematic Reviews and Meta-analysis were followed strictly in conducting the methods and analysis [20]. The protocol of this meta-analysis was prospectively registered on PROSPERO (CRD42022329306).

### Search strategy

We searched the following electronic medical databases: PubMed, Scopus, Web of Science, Google Scholar, and Cochrane Library from inception till December 2022 using the following query: (“Tranexamic acid” OR TXA OR AMCHA OR t-AMCHA OR AMCA OR Anvitoff OR Cyklokapron OR Ugurol OR Spotof OR Transamin OR Amchafibrin OR Exacyl) AND (“Postpartum hemorrhage” OR PPHge). Our electronic search strategy was designed and validated using the Peer Review of Electronic Search Strategies (PRESS) checklist tool [21].

### Eligibility criteria

We included studies that satisfy the following criteria:

- Population: Pregnant women of any age who have not yet developed PPH, either delivered vaginally or by caesarean section.
- Intervention: TXA alone or combined with oxytocin.
- Comparator: placebo or other standard treatment such as oxytocin alone.
- Outcomes: The primary outcome was total blood loss, and occurrence of nausea or/and or vomiting. Secondary outcomes included change in haemoglobin, and PPH occurrence. Additionally, uterotonic agent use, postoperative blood loss, intra-operative blood loss (in case of prophylaxis use), and the incidence of hysterectomy.
- Study design: double arm randomized controlled trials (RCTs).

We excluded studies that were reviews, single-arm studies, conference abstracts, case reports, case series, and studies that assessed total blood loss after any surgery or condition not specific for birth throughout. Moreover, non-English articles and studies that assess irrelevant outcomes were excluded.

### Selection of studies

Retrieved records from the four-database search were screened in a two-step manner. The first step was the title and abstract screening. Then, the full text of articles with eligible abstracts was examined to assess the reliability of their data for meta-analysis and their eligibility for inclusion in the systematic review. The screening was done by three independent authors, and any disagreements were resolved by a fourth author.

### Data extraction

Four reviewers independently extracted relevant data from the included studies using an online data extraction form, that was developed a priori, including 1) study design, 2) characteristics of the study population, 3) risk of bias scopes 4) study outcomes: total blood loss, change in haemoglobin (g/dl), and PPH occurrence. In addition, uterotonic agents used postoperative blood loss, intra-operative blood loss, the occurrence of nausea or/and vomiting, and the incidence of hysterectomy. The differences were resolved through discussions by a fifth author.

### Quality assessment

The quality assessment of included studies was performed by two authors independently using the ROB-II tool for RCTs [22]. The Cochrane tool for evaluating the possibility of bias comprises the subsequent areas: (1) Random sequence generation (selection bias), (2) allocation concealment (selection bias), (3) blinding of participants and personnel (performance bias), (4) blinding of outcome assessment (detection bias), (5) incomplete outcome data (attrition bias), (6) selective reporting (reporting bias) and (7) other potential sources of bias. The authors’ decision is classified as Unclear risk, Low risk, or High risk of bias. The conflicts were solved by the third author.

### Data synthesis

For dichotomous data, the frequency of events and the total number of patients in each group were pooled as the odds ratio between the two groups in the inverse variance method with the random-effects model. While in continuous data, the mean difference (MD) between the two groups from the baseline to endpoint, with its standard deviation (SD), and the total number of patients in each group were pooled in the inverse variance method with the random-effects model for each efficacy measure. The heterogeneity of studies was examined by visual inspection of the forest plots and assessed by the Cochrane Q and I^2^ tests using RevMan version 5.4 for windows. For heterogeneity testing, a *P* < 0.1 and an I-square > 50% were considered for significant heterogeneity.

### Subgroup analysis

We conducted a subgroup analysis according to the time of administration; before and after surgery for CB while < 5 min or > 5 min from the start of birth throughout for VB. Also, subgroups are based on countries; high-income (HIC), upper-middle-income (UMIC), and lower-middle-income countries (LMIC). *P* values less than 0.05 are often reported as ‘statistically significant’ and interpreted as being small enough to justify rejection of the null hypothesis. For heterogeneity testing, a *P* < 0.1 and an I-square > 50% were considered for significant heterogeneity.

### Sensitivity analysis

We performed a leave-one-out model excluding one study at a time to ensure that the overall effect size was not heavily influenced by any single study. Moreover, we analyzed the studies according to their quality (Low risk of bias, some concern, and High risk of bias).

### Reporting bias assessment

We constructed funnel plots to explore the publication bias among studies. Egger’s regression test was used to assess evidence of publication bias [23].

## Results

### Results of literature search and study characteristics

**Fig. 1:**
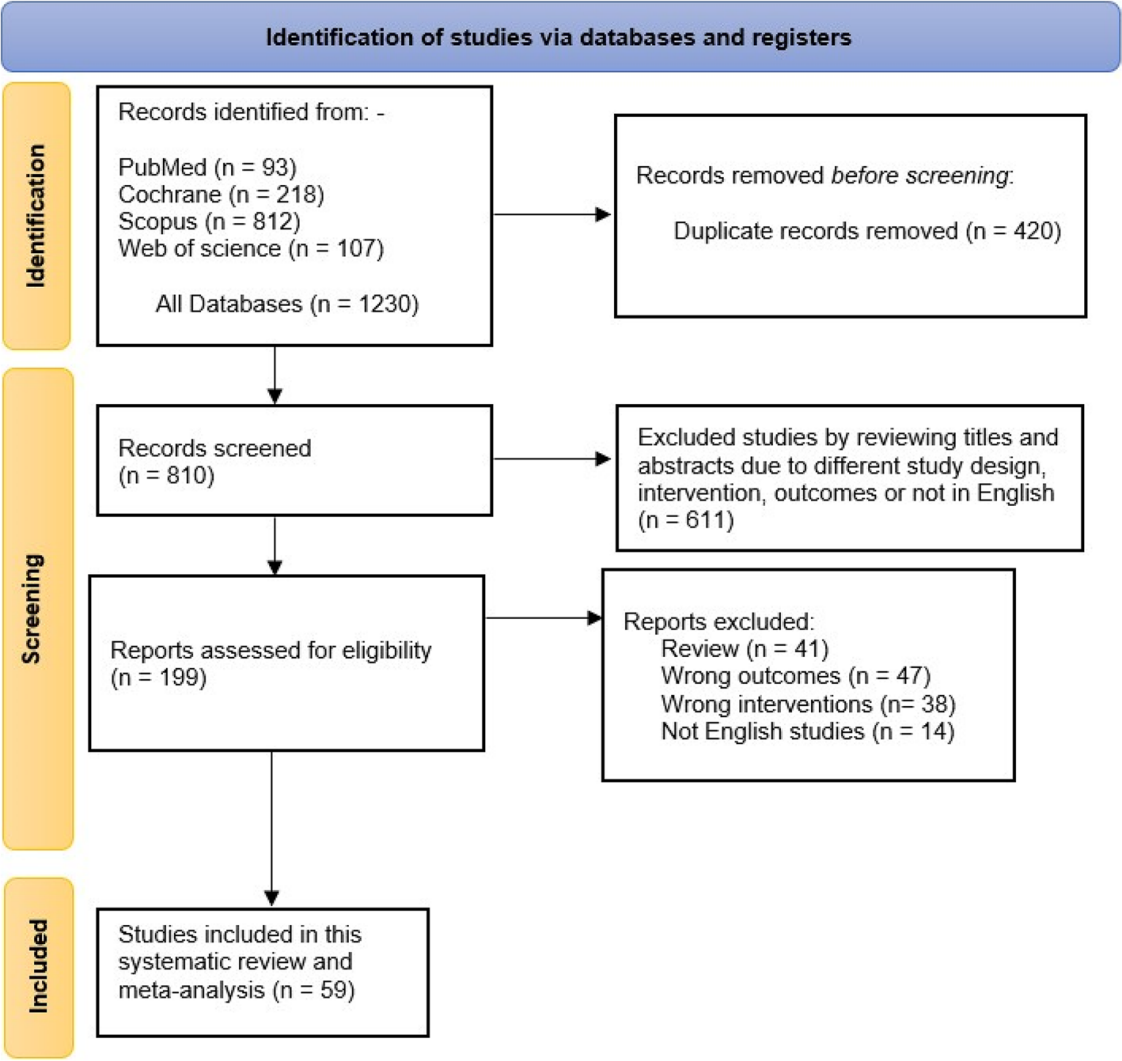
PRISMA flow diagram of studies’ screening and selection

The initial search retrieved 1597 unique records, and 438 were excluded for duplicate records. After title and abstract screening, 960 records are excluded. The full text of 199 studies was then retrieved for the detailed assessment. Finally, 59 studies were included in this systematic review and meta-analysis [6, 7, 12, 18, 24–78]. The references of the included studies were manually searched, and no further articles were included. A flowchart of the study selection process is shown in Fig. 1. A summary of the included studies, their design, and main findings are shown in Supplementary Table 1, while the population characteristics of the included articles are shown in Supplementary Table 2.

### Risk of bias assessment

The overall risk of bias among the included studies varies from low to high risk of bias. The results revealed that 11 studies had a low risk of bias, 27 had some concern risk of bias, and 21 had a high risk of bias, according to the Cochrane risk of bias tool 2. Details of the risk of bias assessment are shown in Supplementary Table 3.

### Primary outcomes

#### Total blood loss

**Fig. 2:**
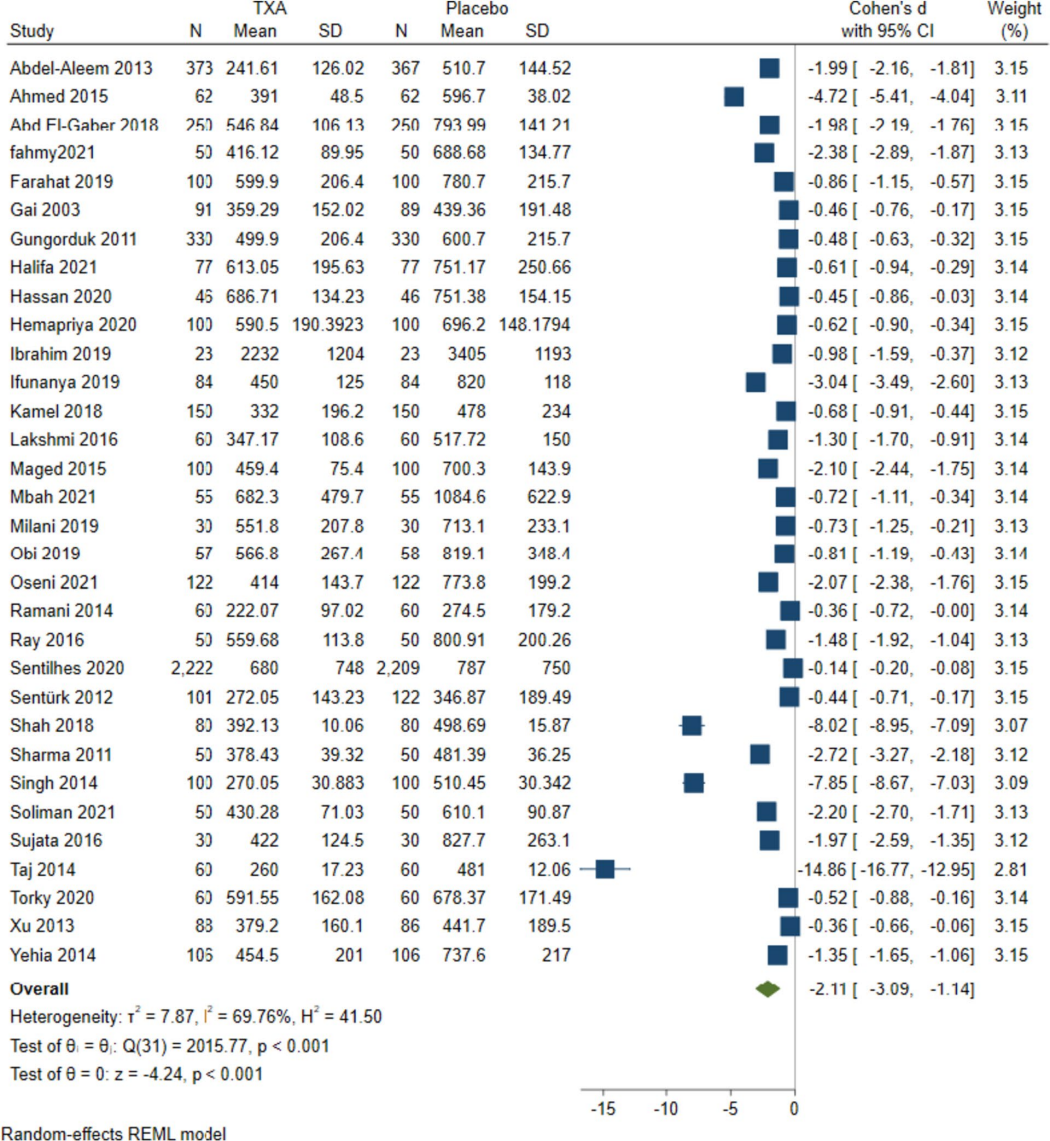
Forest plots of mean difference in total blood loss in CB

The overall effect statistically favored TXA over the placebo for both CB (MD= -2.11 mL, 95%CI [-3.09 to -1.14], *P* < 0.001); heterogeneity was high (I^2^ = 69.76%, *P* < 0.001) Fig. 2 and VB (MD= -0.89 mL, 95%CI [-1.47 to -0.31], *P* = 0.01); with high heterogeneity (I^2^ = 68.23%, *P* < 0.001) Fig. 5A. Moreover, similar results were obtained during the subgroup analysis. For CB, we observed that TXA was favored over placebo in reducing total blood loss regardless of (i) the time of administration (before or after the surgery), (ii) the quality of included studies, or (iii) the country’s income level.

For VB, TXA was favored over placebo in reducing total blood loss if (i) administered over 5 min from the VB time, (ii) in low-risk and high-risk studies, and (iii) only in LMIC countries (*P* values were not applicable for HIC or UMIC) Table 1.

**Table 1:** Shows the results of the subgroups analysis

**Time of administration**				
	***CB***		***VB***	
	***before surgery***	***after surgery***	** < *5 min***	** > *5 min***
***Total blood loss***	MD = -2.29 mL, 95%CI [-3.49 to -1.10], *P* < 0.001; (I^2^ = 69.72%) [7, 24–26, 29, 32, 34, 37, 38, 45, 47–49, 54–57, 61–65, 68–70, 75]	MD = -1.38 mL, 95%CI [-2.29 to -0.48], *P* < 0.001; (I^2^ = 48.08%) [31, 39–41, 60, 71]	MD = -0.62 mL, 95%CI [-1.39 to 0.14], *P* = 0.65; (I^2^ = 67.58%) [18, 66, 73, 77]	MD = -1.21 mL, 95%CI [-2.10 to -0.32], *P* = 0.02; (I^2^ = 57.18%) [6, 27, 42, 50, 74, 76]
***Change in HB***	MD = 0.84, 95%CI [0.68 to 0.98], *P* < 0.001; (I^2^ = 64.67%) [24–26, 29, 32, 34, 38–41, 45, 48, 49, 54–57, 61–65, 68–70, 75]	MD = 1.32, 95%CI [1.21 to 2.26], *P* < 0.001; (I^2^ = 58.12%) [7, 31, 37, 47, 60, 71]	MD = -0.05%, 95%CI [-0.49 to 0.39], *P* = 0.65; (I^2^ = 61.95%) [18, 46, 77]	MD = 0.42%, 95%CI [0.21 to 0.63], *P* < 0.001; (I^2^ = 45.88%) [36, 42, 76]
***Occurrence of PPH***	OR = 0.28 mL, 95%CI [0.18 to 0.44], *P* < 0.001; (I^2^ = 58.65%) [24, 26, 28, 29, 34, 37, 47, 48, 54, 55, 64, 65, 69, 70, 75]	OR = 0.45 mL, 95%CI [0.29 to 0.72], *P* < 0.001; (I^2^ = 66.82%) [12, 35, 39, 41, 43, 52, 53, 60, 71]	OR = 0.81, 95%CI [0.65 to 1.01], *P* = 0.57; (I^2^ = 0%) [18, 46, 66, 77]	OR = 0.42, 95%CI [0.23 to 0.75], *P* = 0.01; (I^2^ = 30.65%) [27, 36, 50, 58, 74, 76]
**Quality of included studies**				
		***Low risk of bias***	***Some concern***	***High risk of bias***
***Total blood loss***	CB	MD = -0.75 mL, 95%CI [-1.28 to -0.22], *P* < 0.001; (I^2^ = 58.19%) [25, 34, 37, 38, 54, 60]	MD = -1.58 mL, 95%CI [-2.18 to -0.98], *P* < 0.001; (I^2^ = 67.45%) [24, 26, 31, 39–41, 47, 49, 55, 61, 64, 65, 69–71]	MD = -3.66 mL, 95%CI [-6.33 to -1.00], *P* < 0.001; (I^2^ = 69.80%) [7, 29, 32, 45, 48, 56, 57, 62, 63, 68, 75]
	VB	(MD = -0.51 mL, 95%CI [-0.85 to -0.16], *P* = 0.01), high heterogeneity (I^2^ = 61.91%) [30, 42, 50, 73, 76, 77]	MD = -0.38 mL, 95%CI [-1.00 to -0.24], *P* = 0.65; (I^2^ = 57.72%) [66, 74]	MD = -1.96 mL, 95%CI [-3.50 to -0.41], *P* < 0.001; (I^2^ = 67.88%) [6, 18, 27]
***Change in HB***	CB	MD = 0.66%, 95%CI [0.29 to 0.97], *P* < 0.001; (I^2^ = 54.39%) [32, 48, 49, 60]	MD = 1.13%, 95%CI [1.05 to 1.36], *P* < 0.001; (I^2^ = 62.03%) [24–26, 29, 31, 37–39, 45, 47, 54, 61, 64, 65, 69–71, 75]	MD = 1.27, 95%CI [1.12 to 1.98], *P* < 0.001; (I^2^ = 57.78%) [7, 34, 40, 41, 55–57, 62, 63, 68]
	VB	MD = 0.32, 95%CI [0.02 to 0.62], *P* = 0.01; (I^2^ = 65.05%) [42, 46, 50, 77]	MD = 0.31, 95%CI [0.14 to 0.49], *P* = 0.01; (I^2^ = 0%) [36, 76]	MD = -0.50, 95%CI [-0.81 to -0.18] [18]
***Occurrence of PPH***	CB	OR = 0.52 mL, 95%CI [0.33 to 0.83], *P* < 0.001; (I^2^ = 72.55%) [34, 37, 52, 54, 60]	OR = 0.30 mL, 95%CI [0.19 to 0.46], *P* < 0.001; (I^2^ = 52.33%) [24, 26, 28, 35, 39, 41, 43, 47, 53, 55, 64, 65, 69–71]	OR = 0.21 mL, 95%CI [0.07 to 0.59], *P* = 0.001; (I^2^ = 52.33%) [12, 29, 48, 75]
	VB	OR = 0.80, 95%CI [0.65 to 0.99], *P* = 0.04; (I^2^ = 0%) [46, 50, 76, 77]	OR = 0.26, 95%CI [0.13 to 0.54], *P* < 0.001; (I^2^ = 0%) [36, 58, 66, 74]	OR = 0.60, 95%CI [0.24 to 1.48], *P* = 0.70; (I^2^ = 0%) [18, 27]
**Countries**				
		***HIC***	***UMIC***	***LMIC***
***Total blood loss***	CB	MD = -0.28 mL, 95%CI [-0.49 to -0.07], *P* = 0.04; (I^2^ = 64.18%) [34, 60, 70]	MD = -0.47 mL, 95%CI [-0.60 to -0.33], *P* = 0.02; (I^2^ = 0.00%) [37, 61]	MD = -2.44 mL, 95%CI [-3.57 to -1.32], *P* < 0.001; (I^2^ = 69.62%) [7, 24–26, 29, 31, 32, 38–41, 45, 47–49, 54–57, 62–65, 68, 69, 71, 75]
	VB	MD = -0.06, 95%CI [-0.12 to 0.01] [77]	_	MD = -0.98 mL, 95%CI [-1.59 to -0.36], *P* < 0.001; (I^2^ = 67.03%) [6, 18, 27, 30, 42, 50, 66, 73, 74, 76]
***Change in HB***	CB	MD = 0.60, 95%CI [-0.27 to 1.47], *P* = 0.51; (I^2^ = 56.59%) [60, 70]	MD = 0.50, 95%CI [0.03 to 0.97], *P* = 0.01; (I^2^ = 59.25%) [32, 61]	MD = 1.12, 95%CI [1.02 to 1.38], *P* < 0.001; (I^2^ = 65.22%) [7, 24–26, 29, 31, 34, 37–41, 45, 47, 48, 54–57, 62–65, 68, 69, 71, 75]
	VB	MD = 0.02, 95%CI [-0.05 to 0.08] [77]	MD = 0.31, 95%CI [0.14 to 0.49], *P* = 0.01; (I^2^ = 0%) [36, 76]	MD = 0.21, 95%CI [-0.28 to 0.71], *P* = 0.65; (I^2^ = 70.47%) [18, 42, 46, 50]
***Occurrence of PPH***	CB	OR = 0.81, 95%CI [0.61 to 1.07], *P* = 0.28; (I^2^ = 33.13%) [34, 60, 70]	OR = 0.37, 95%CI [0.15 to 0.89] [37]	OR = 0.27, 95%CI [0.19 to 0.38], *P* < 0.001; (I^2^ = 39.65%) [12, 24, 26, 28, 29, 35, 39, 41, 43, 47, 48, 52–55, 64, 65, 69, 71, 75]
	VB	MD = 0.83, 95%CI [0.66 to 1.03] [77]	MD = 0.25, 95%CI [0.09 to 0.67] [36]	OR = 0.53, 95%CI [0.32 to 0.85], *P* = 0.03; (I^2^ = 6.90%) [18, 27, 46, 74, 76]

*Abbreviations: VB* Vaginal birth, *CB* Cesarean birth, *MD* Mean Difference, *OR* Odd ratios, *CI* Confidence intervals, *HIC* High-income countries, *UMIC* Upper middle-income countries, *LMIC* Lower middle-income countries, *HB* Hemoglobin, *PPH* Post-partum hemorrhage

#### Nausea/vomiting

The pooled effect estimated showed that TXA was associated with higher odds regarding nausea and/or vomiting in women who had VB and CB, with the following values (OR = 2.36, 95% CI [1.32 to 4.21], *P* = 0.02, (OR = 1.36, 95% CI [1.07 to 1.74], *P* = 0.01), respectively, Table 2.

**Table 2:** Shows the results of the analysis of the secondary outcomes

Outcomes	CB	VB
***The additional use of uterotonic agents***	OR = 0.46, 95%CI [0.34 to 0.62], *P* = 0.01; (I^2^ = 52.53%) [24, 25, 29, 33, 37, 38, 41, 48, 53, 54, 60–62, 65]	OR = 0.55, 95%CI [0.37 to 0.80], *P* = 0.02; (I^2^ = 45.62%) [18, 27, 30, 36, 42, 46, 50, 58, 66, 76, 77]
***Postoperative blood loss***	MD= -1.54 mL, 95%CI [-2.02 to -1.06], *P* = 0.01; (I^2^ = 67.76%) [12, 24, 26, 28, 33, 34, 38, 39, 44, 45, 49, 51–53, 56, 57, 59, 62–64, 67, 70–72, 75]	MD= -0.55 mL, 95%CI [-0.73 to -0.37], *P* = 0.01; (I^2^ = 20.91%) [36, 50, 78]
***Intra-operative blood loss***	MD= -1.96 mL, 95%CI [-2.77 to -1.15], *P* < 0.001; (I^2^ = 69.15%) [12, 24, 26, 28, 33–35, 38, 39, 44, 45, 49, 51–53, 56, 57, 62–64, 67, 70–72, 75]	MD = 0.05 mL, 95%CI [-0.21 to 0.31], *P* = 0.70; (I^2^ = 33.51%) [30, 50]
***The incidence of hysterectomy***	OR = 1.59, 95%CI [0.71 to 3.53], *P* = 0.26; (I^2^ = 0.00%) [25, 37, 53, 54, 60, 64, 69]	OR = 0.34, 95%CI [0.07 to 1.75], *P* = 0.20; (I^2^ = 0%) [27, 30, 78]
***Occurrence of nausea/vomiting***	OR = 1.36, 95%CI [1.07 to 1.74], *P* = 0.01; (I^2^ = 12.63%) [12, 31, 32, 35, 38, 43, 52–54, 57, 60, 69–71]	OR = 2.36, 95%CI [1.32 to 4.21], *P* = 0.02; (I^2^ = 67.19%) [27, 30, 36, 50, 66, 74, 77, 78]

*Abbreviations:* *VB* Vaginal birth, *CB* Cesarean birth, *MD* Mean Difference, *OR* Odd ratios, *CI* Confidence intervals

### Secondary outcomes

#### Change in haemoglobin (g/dl)

**Fig. 3:**
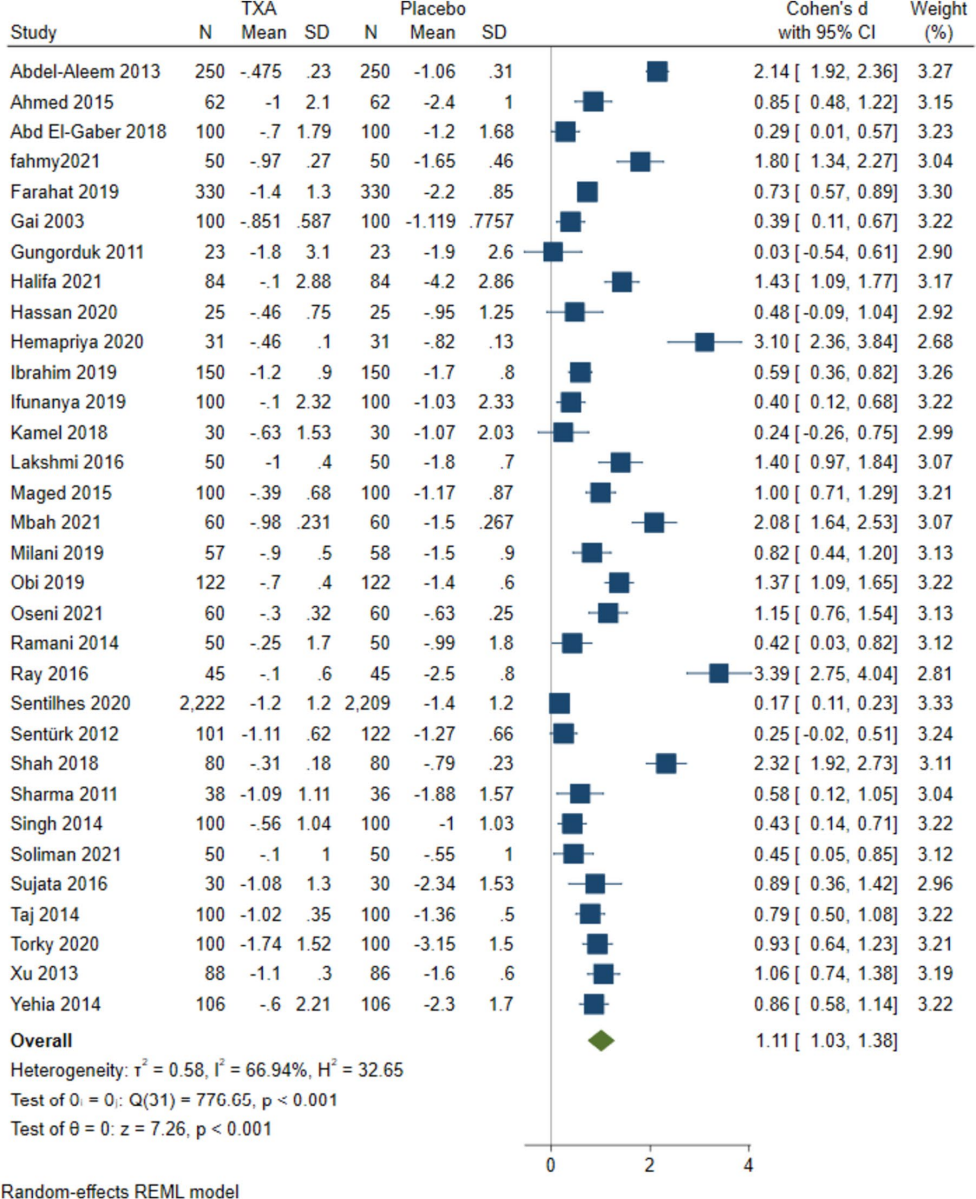
Forest plots of mean difference in change in HB in CB

 TXA significantly reduced change in haemoglobin (g/dl) more than the placebo for CB (MD = 1.11, 95%CI [1.03 to 1.38], *P* < 0.001); the estimated heterogeneity was high (I^2^ = 66.94%, *P* < 0.001), Fig. 3. On the other hand, the usage of TXA in VB was not favoured in reducing the change in haemoglobin (g/dl) in comparison with placebo (MD = 0.20 g/dl, 95%CI [-0.07 to 0.48], *P* = 0.15), heterogeneity was high (I^2^ = 70.38%, *P* = 0.32) Fig. 5B. Subgroup analysis indicated that the association remained significant in all subgroups of administration time, quality of included studies, and countries except HIC while for VB insignificant findings were in administration in < 5 min and LMIC subgroups Table 1.

#### Occurrence of PPH

**Fig. 4:**
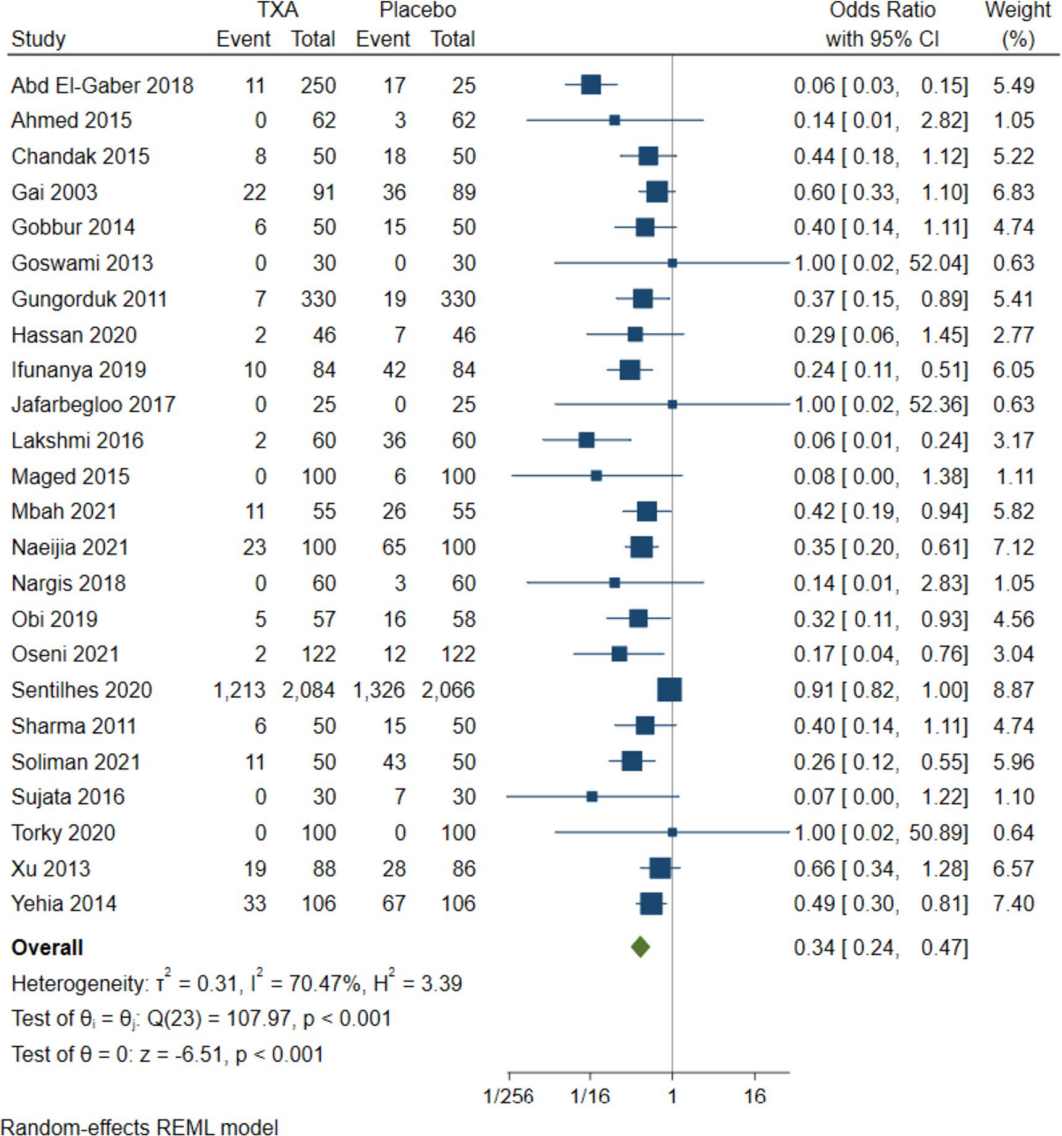
Forest plot of odd ratios in occurrence of PPH in CB

**Fig. 5:**
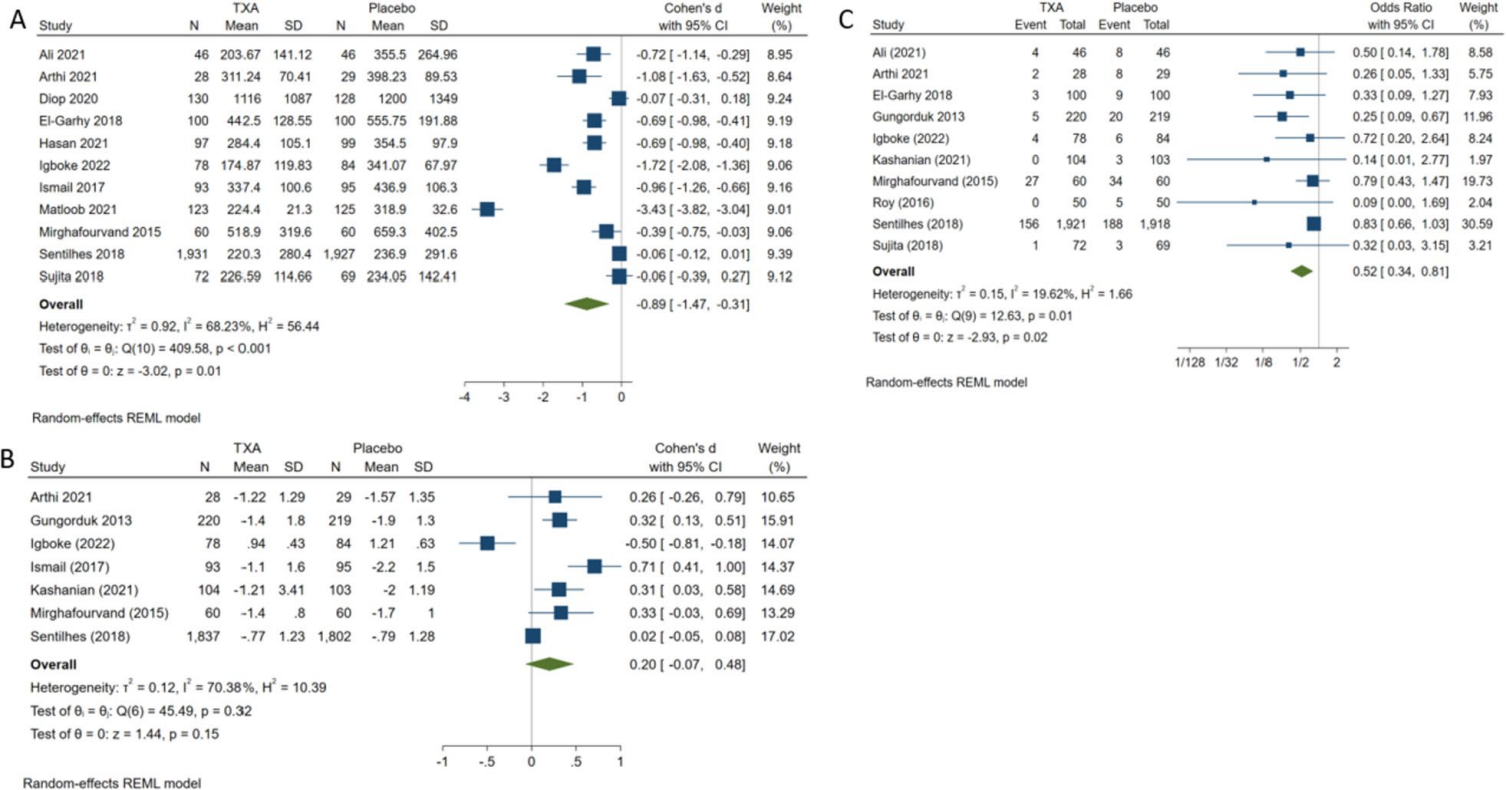
Forest plots of mean difference in **A** total blood loss in VB, **B** Change in HB in VB, and Forest plot of odd ratios in **C** Occurrence of PPH in VB

Analysis of the pooled studies retrieved significant efficacy of TXA over placebo on occurrence rates of PPH for CB (OR = 0.34, 95%CI [0.24 to 0.47], *P* < 0.001); high heterogeneity (I^2^ = 70.47%, *P* < 0.001) Fig. 4, and VB (OR = 0.52, 95%CI [0.34 to 0.81], *P* = 0.02), low heterogeneity (I^2^ = 19.62%, *P* = 0.01) Fig. 5C. Subgroup analysis indicated that the association remained significant in all subgroups of administration time, quality of included studies, and countries except HIC for VB insignificant findings were in administration in < 5 min and high-risk quality subgroups Table 1.

#### Publication bias

Visual inspection of funnel plots in terms of total blood loss, change in hemoglobin, and occurrence rate of PPH revealed asymmetry. So, there was evidence of potential publication bias Supplementary Figure 1.

### Other outcomes

For CB groups, fewer use of additional uterotonic agents occurred in the TXA than placebo group (OR = 0.46, 95%CI [0.34 to 0.62], *P* = 0.01), postoperative blood loss (MD=-1.54, 95%CI [-2.02 to -1.06], *P* = 0.01), and intra-operative blood loss (MD=-1.96, 95%CI [-2.77 to -1.15], *P* < 0.001). While the incidence of hysterectomy showed insignificant results (OR = 1.59, 95%CI [0.71 to 3.35], *P* = 0.26). On the other hand, the overall efficacy of VB groups significantly favoured TXA over placebo with the additional use of uterotonic agents (OR = 0.55, 95%CI [0.37 to 0.80], *P* = 0.02), and post-operative blood loss (MD=-0.55, 95%CI [-0.73 to -0.37], *P* = 0.01). While the incidence of hysterectomy and intra-operative blood loss showed insignificant results (OR = 0.34, 95%CI [0.07 to 1.75], *P* = 0.20), (MD = 0.05, 95%CI [-0.21 to 0.31], *P* = 0.70), respectively, Table 2.

We further performed a meta regression analysis based on the type of mode of birth throughout, to study whether the effect of TXA is proportional to the mode of birth throughout and figured that there was no association between the effect estimate and mode of birth throughout in term of total blood loss (CB *P* < 0.001, VB *P* = 0.01), occurrence rate of PPH(CB *P* < 0.001, VB *P* = 0.02), the additional use of uterotonic agents(CB *P* = 0.01, VB *P* = 0.02), post-operative blood loss (CB *P* = 0.01, VB *P* = 0.01) and the occurrence of nausea and/or vomiting(CB *P* = 0.01, VB *P* = 0.01).

### Quality of evidence

The overall quality of evidence was high for the occurrence of nausea/ vomiting and the incidence of hysterectomy for both CB and VB groups. Also, the high quality was assessed for VB groups of occurrences of PPH, postoperative blood loss, and additional uterotonic agents. On the other hand, moderate evidence quality was reported for the rest of the outcomes mostly due to inconsistency. Details of each domain in the GRADE assessment are reported in Table 3.

**Table 3:** Shows the assessment of the certainty in evidence using the GRADE framework

***Outcome***	***Mode of birth throughout***	***Effect Size, 95% CI, and P value***	***Number of studies***	***Study Design***	***Risk of Bias***	***Inconsistency***	***Indirectness***	***Imprecision***	***Others ***	***Evaluation***
*Total blood loss*	*CB*	*MD 2.11, 95% CI [-3.09 to -1.14], P* < *0.001* [7, 24–26, 29, 31, 32, 34, 37–41, 45, 47–49, 54–57, 60–65, 68–71, 75]	*32*	*RCT*	*No downgrade*	*The downgrade by one level*	*No downgrade*	*No downgrade*	*None*	*Moderate*
	*VB*	*MD -0.89, 95%CI [-1.47 to -0.31], P* = *0.01* [6, 18, 27, 30, 42, 50, 66, 73, 74, 76, 77]	*11*	*RCT*	*No downgrade*	*The downgrade by one level*	*No downgrade*	*No downgrade*	*None*	*Moderate*
*Occurrence of PPH*	*CB*	*OR 0.34, 95%CI [0.24 to 0.47], P* < *0.001* [12, 24, 26, 28, 29, 34, 35, 37, 39, 41, 43, 47, 48, 52–55, 60, 64, 65, 69–71, 75]	*24*	*RCT*	*No downgrade*	*The downgrade by one level*	*No downgrade*	*No downgrade*	*None*	*Moderate*
	*VB*	*OR 0.52, 95%CI [0.34 to 0.81], P* = *0.02* [18, 27, 36, 46, 50, 58, 66, 74, 76, 77]	*10*	*RCT*	*No downgrade*	*No downgrade*	*No downgrade*	*No downgrade*	*None*	*High*
*Mean change in Hb*	*CB*	*MD 1.11, 95%CI [1.03 to 1.38], P* < *0.001* [7, 24–26, 29, 31, 32, 34, 37–41, 45, 47–49, 54–57, 60–65, 68–71, 75]	*32*	*RCT*	*No downgrade*	*The downgrade by one level*	*No downgrade*	*No downgrade*	*None*	*Moderate*
	*VB*	*MD 0.20, 95%CI [-0.07 to 0.48], P* = *0.15* [18, 36, 42, 46, 50, 76, 77]	*7*	*RCT*	*No downgrade*	*The downgrade by one level*	*No downgrade*	*No downgrade*	*None*	*Moderate*
*Intraoperative blood loss*	*CB*	*MD -1.96, 95%CI [-2.77 to -1.15], P* < *0.001* [12, 24, 26, 28, 33–35, 38, 39, 44, 45, 49, 51–53, 56, 57, 62–64, 67, 70–72, 75]	*25*	*RCT*	*The downgrade*	*Downgrade by one level*	*No downgrade*	*No downgrade*	*None*	*Moderate*
	*VB*	*MD 0.05 mL, 95%CI [-0.21 to 0.31], P* = *0.70* [30, 50]	*2*	*RCT*	*No downgrade*	*No downgrade*	*No downgrade*	*The downgrade by one level*	*None*	*Moderate*
*Postoperative blood loss*	*CB*	*MD -1.54, 95%CI [-2.02 to -1.06], P* = *0.01* [12, 24, 26, 28, 33, 34, 38, 39, 44, 45, 49, 51–53, 56, 57, 59, 62–64, 67, 70–72, 75]	*25*	*RCT*	*No downgrade*	*The downgrade by one level*	*No downgrade*	*No downgrade*	*None*	*Moderate*
	*VB*	*MD -0.55 mL, 95%CI [-0.73 to -0.37], P* = *0.01* [36, 50, 78]	*3*	*RCT*	*No downgrade*	*No downgrade*	*No downgrade*	*No downgrade*	*None*	*High*
*Additional uterotonic agent*	*CB*	*OR 0.46, 95%CI [0.34 to 0.62], P* = *0.01* [24, 25, 29, 33, 37, 38, 41, 48, 53, 54, 60–62, 65]	*14*	*RCT*	*No downgrade*	*The downgrade by one level*	*No downgrade*	*No downgrade*	*None*	*Moderate*
	*VB*	*OR 0.55, 95%CI [0.37 to 0.80], P* = *0.02* [18, 27, 30, 36, 42, 46, 50, 58, 66, 76, 77]	*11*	*RCT*	*No downgrade*	*No downgrade*	*No downgrade*	*No downgrade*	*None*	*High*
*Occurrence of nausea or/and vomiting*	*CB*	*OR 1.36, 95%CI [1.07 to 1.74], P* = *0.01* [12, 31, 32, 35, 38, 43, 52–54, 57, 60, 69–71]	*14*	*RCT*	*No downgrade*	*No downgrade*	*No downgrade*	*No downgrade*	*None*	*High*
	*VB*	*OR 2.36, 95%CI [1.32 to 4.21], P* = *0.02* [27, 30, 36, 50, 66, 74, 77, 78]	*8*	*RCT*	*No downgrade*	*Downgrade by one*	*No downgrade*	*No downgrade*	*None*	*High*
*Incidence of hysterectomy*	*CB*	*OR 1.59, 95%CI [0.71 to 3.53], P* = *0.26* [25, 37, 53, 54, 60, 64, 69]	*7*	*RCT*	*No downgrade*	*No downgrade*	*No downgrade*	*No downgrade*	*None*	*High*
	*VB*	*OR 0.34, 95%CI [0.07 to 1.75], P* = *0.20* [27, 30, 78]	*3*	*RCT*	*No downgrade*	*No downgrade*	*No downgrade*	*No downgrade*	*None*	*High*

Others are publication bias, large effect, dose–response, and plausible confounding factors

High indicates that we are extremely certain that the actual effect is close to the effect estimate

Moderate indicates that the impact estimate has moderate confidence: the actual effect is likely to be close

Low indicates that the confidence about the result is limited and the true effect can be different from our result

*RCTs* Randomized Control Trials, *CI* Confidence Interval

## Discussion

This meta-analysis of 59 RCTs comprising 18,649 participants indicated that the administration of TXA is associated with significantly lower total blood loss, the occurrence of PPH, the additional use of uterotonic agents, and the occurrence of nausea or/and vomiting in women undergoing caesarean or vaginal birth. The usage of TXA for the reduction of haemoglobin change and post-operative and intra-operative blood loss was favoured in CB, not VB groups. The incidence of hysterectomy showed insignificant results in both CB and VB.

We reported similar results to previous meta-analyses done for the CB mode of birth throughout. The Cochrane systematic review of 9 trials (*N* = 2453) by Novikova et al. has indicated a significant decrease in PPH with TXA as a prophylaxis treatment – a moderate quality of evidence [79]. Also, the meta-analysis of Li et al. [11] included 15 studies with a total of 3353 patients, showed a significant decrease in total blood loss with TXA use (MD = 154.25 mL). A recent meta-analysis confirmed these results (21 studies, 3852 participants) [80]. Therefore, our meta-analysis confirmed and extended previous studies’ results by including a significantly larger sample size despite strict eligibility criteria, enabling the exploration of heterogeneity and a more accurate appraisal of evidence quality.

In the contemporary evidence supported by previous systematic reviews and meta-analyses for VB mode, Saccone et al. [81] (S = 4 RCTs [77, 82–84], *N* = 4,671) reported that prophylactic administration of TXA significantly decreased the rate of PPH and total blood loss compared with placebo. Insignificant results were obtained for blood transfusion rate and average postoperative haemoglobin and haematocrit levels. Regarding the side effects, the rates of nausea and vomiting were significantly higher in favour of the placebo. Moreover, Della Corte et al. [85] Conducted a systematic review and meta-analysis of only two RCTs [78, 86] (*N* = 14,363 patients) and reported that TXA significantly reduced the rate of hysterectomy compared with placebo. Nevertheless, the maternal death rates (all causes), blood transfusion, and admission to the intensive care unit were insignificant between groups.

Notably, we conducted a comparison table to summarise our results head-to-head with the two most recent systematic reviews that reported the efficacy of TXA on PPH [87, 88]. All data are presented in Table 4. Methodologically, the previous studies have shown some limitations in the number of included studies and patients that were used in comparison to our study, Bellos et al. [87] and Abo-Zaid et al. [88] Included 36, 16 studies and 10,659, 7122 participants respectively. However, we included 59 trials and 18,649 patients in the analysis.

**Table 4:** Comparison of our study with previously (recently) published systematic reviews and meta-analyses

	*Bellos 2022* [87]		*Abu-Zaid 2022* [88]	*Our study*	
*Number of studies*	*36*		*16*	*59*	
*Number of patients*	*10,659*		*7122*	*18,649*	
*The design of studies included*	*RCTs*		*RCTs*	*RCTS*	
*Mode of birth throughout*	*CB*		*VB*	*CB and VB*	
*Total blood loss*	*Favor TXA*	*Moderate evidence*	*Favor TXA*	*Favor TXA*	*Moderate evidence*
*Change in hemoglobin*	*Favor TXA*	*Moderate evidence*	*Favor TXA*	*Favor TXA (CB only)*	*Moderate evidence*
*The occurrence rate of PPH*	*Favor TXA*	*Low evidence*	*Favor TXA*	*Favor TXA*	*Moderate for CB, High for VB*
*Additional uterotonic agents*	*Favor TXA*	*Low evidence*	*Favor TXA*	*Favor TXA*	*Moderate for CB, High for VB*
*Intraoperative blood loss*	*NR*		*NR*	*Favor TXA (CB only)*	*Moderate evidence*
*Post-operative blood loss*	*NR*		*NR*	*Favor TXA*	*Moderate for CB, High for VB*
*Occurrence of nausea and vomiting*	*NR*		*Not favor TXA*	*Not Favor TXA*	*High evidence*
*Incidence of hysterectomy*	*NR*		*NR*	*NS*	*High evidence*

*TA* Tranexamic acid, *RCTs* Randomized clinical trials, *NR* Not reported, *NS* Not significant

Regarding the analysed outcomes and their quality of evidence, TXA was associated with a lower risk of total blood loss and a positive effect on levels of haemoglobin with moderate quality of evidence in Bellos et al. [87] and our study. TXA was favoured also by Abo-Zaid et al. [88]; however, they did not assess the quality of evidence. in study done by Bellos et al. [87], TXA was associated with lower incidence of PPH rate and the need for additional uterotonic agents, with low-quality of evidence. Compared to our study, there was moderate quality evidence for CB, and High quality of evidence for VB, while Abo-Zaid et al. [88] did not assess the quality of evidence.

On the other hand, the incidence of nausea or/and vomiting was comparable in TXA and placebo in Abo-Zaid et al. [88]. Similar to our results with high-quality evidence. Nevertheless, TXA was superior to placebo regarding intra and postoperative blood loss, with moderate quality of evidence, moderate for CB, and high for VB respectively. While the incidence of hysterectomy didn’t favor TXA in our study, despite the high quality of evidence, the results were not statistically significant. Bellos et al. and Abo-Zaid et al. [87, 88] did not assess these three outcomes.

### Future perspective

TXA is an important medication that should be used to treat PPH as per WHO recommendations. We have emphasized the preventive use of TXA. Its benefits in reducing bleeding and preventing further complications make it a crucial tool in managing this serious condition. It is important for healthcare providers to carefully consider the risks and benefits of using tranexamic acid on a case-by-case basis. In the future, further research may be conducted to explore the optimal dosing and timing of TXA administration in PPH management. Additionally, efforts may be made to increase access to this medication in low-resource settings where PPH is more prevalent.

#### Strength and limitations

This study has several strength points: (1) to the best of our knowledge, this systematic review and meta-analysis of TXA for treatment or prophylaxis of PPH provides the most comprehensive evidence to date, (2) the protocol of this study was prospectively registered and all steps were conducted in strict accordance with the PRISMA statement guidelines as well as Cochrane Handbook of Systematic Review and Meta-analysis, (3) the certainty in evidence was evaluated by the GRADE framework, (4) the included studies were classified according to multiple considerations such as time of TXA administration and quality of the included studies to provide the most updated and comprehensive evidence to guide the further research.

The major limitation of our meta-analysis is that we excluded many studies due to their low quality (duplicated/fabricated results). Additionally, different techniques were applied to estimate the blood loss, which is difficult to pool in our study, especially during internal bleeding. There is also variation in administering/co-administration of oxytocin and TXA doses pre-or postoperative or during the operation.

Our review is further limited due to minimal assessment or reporting of potential harm effects of TXA compared to the benefits in pregnant women. This is because few studies were identified and included that measured adverse effects, for example, thromboembolism. Thus it is difficult to balance the benefits with harms in our findings.

Although included studies have not shown a significant increase in these events with TXA use, caution should still be exercised when administering it to women with pre-existing clotting disorders or those at high risk for thromboembolism.

## Conclusions

Ultimately, this meta-analysis suggests that TXA is effective and safe in preventing PPH when administered promptly and appropriately. Nonetheless, further research is required to determine optimal dosing regimens and to identify any potential long-term risks associated with its use in both high and low-risk women.

### Supplementary Information

**Additional file 1: Supplementary Table 1.** Summary of the included studies.

**Additional file 2: Supplementary Table 2.** The characteristics of the included studies’ populations.

**Additional file 3: Supplementary Table 3.** Assessment risk of bias of the included studies.

**Additional file 4: Supplementary Figure 1: **Visual inspection of funnel plots in terms of total blood loss, change in hemoglobin, and occurrence rate of PPH.

## Data Availability

The datasets used and/or analysed during the current study are available from the corresponding author on reasonable request.

## Abbreviations

PPH: Postpartum Hemorrhage
CB: Cesarean Section
VB: Vaginal birth
TXA: Tranexamic Acid
HB: Haemoglobin
HIC: High-income countries
UMIC: Upper-middle-income countries
LMIC: Lower-middle-income countries
RCTs: Randomized Controlled Trials
MD: Mean Differences
SD: Standard Deviation
OR: Odd Ratios
CI: Confidence intervals
