# Supplementary material for: Efficacy and safety of tranexamic acid in prevention of postpartum hemorrhage: a systematic review and meta-analysis of 18,649 patients
Source: BMC Pregnancy Childbirth. 2023 Nov 24;23:817. doi: 10.1186/s12884-023-06100-8 (PMC10668444; doi:10.1186/s12884-023-06100-8)
Supplement: Supplementary file 1 — Additional file 1: Supplementary Table 1. Summary of the included studies. [file 12884_2023_6100_MOESM1_ESM.docx]

**Supplementary Table 1**: Summary of the included studies

| **Ref.** | **Study ID** | **Study design** | **Inclusion criteria** | **Exclusion criteria** | **Study Arms** | | | | **Main findings** | Quality Assessment |
| --- | --- | --- | --- | --- | --- | --- | --- | --- | --- | --- |
|  |  |  |  |  | **Name** | **Duration of administration** | **Name** | **Duration of administration** |  |  |
| (1) | Abdel-Aleem (2013) | RCT | Pregnant women who planned to have elective CS and pregnant women with singleton fetus at 37 weeks gestation were considered potentially eligible for the study. | Women showed one of these criteria:  -History of medical disorders.  -Preeclampsia.  -Antepartum hemorrhage.  -History of thromboembolic disorders.  -Polyhydramnios.  -Macrosomia. -History of sensitivity to TA  -Subjects taking anticoagulant therapy. | Tranexamic Acid | over 10 min | nothing | NA | Subjects who received TA showed significant reduction in the mean blood loss. | Low risk of bias |
| (2) | Ahmed (2015) | RCT | Women with term singleton pregnancy prepared for elective CS. Date of delivery was determined by completed weeks of gestation of reliable menstrual cycle if the last menstrual period (LMP) was certain; if the LMP was not assured, ultrasound data were used. | Women with anticipated excessive blood loss during surgery, i.e.,  -Placenta previa.  -Twin pregnancy. -Presence of uterine fibroid,  Women with history of TA allergy or thromboembolic disorders and those suffering from systemic medical diseases (hepatic, cardiac, renal, etc.) | tranexamic acid plus oxytocin or ergometrine | over 5 min | Oxytocin or ergometrine without TA. | NA | Subjects who received TA showed:  -Lower amount of blood loss.  -Hemoglobin and hematocrit levels were significantly higher. | Some concern |
| (3) | Ali (2021) | RCT | Full-term pregnant women with singleton pregnancy being delivered vaginally,  macrosomia, multiple pregnancy and polyhydramnios. | Women with history of:  -Thrombosis.  -Epilepsy. -Medical problems including the kidney, heart, liver and brain.  -Known allergy to TA.  -Severe medical and surgical complications including the liver, heart or kidney.  -Known hemostatic abnormalities and bleeding disorders before pregnancy. | Tranexamic acid | over 5 min | oxytocin | NA | The use of tranexamic acid could help to:  -Reduce blood loss during delivery.  -Decreases need for uterotonics and hence decreases morbidity and mortality. | High risk of bias |
| (4) | Arthi 2021 | RCT | Full-term pregnant women aged 18 years or more with singleton pregnancy and cephalic presentation delivered vaginally.  . | Women with:  -Hemoglobin ˂8 gm%  -Uterine scar.  -Severe preeclampsia.  -Macrosomia.  -Polyhydramnios.  -IUFD.  -Abnormal placentation.  -Previous history of PPH.  -History of thrombosis or epilepsy.  -Medical problems such as the liver, heart, brain, and kidney.  -Uterine fibroids.  -Hemostatic abnormalities  -Allergy to TXA | Tranexamic acid | Over 30 minutes. | Placebo | Over 5 minutes. | The use of tranexamic acid could help to:  -Reducing blood loss during delivery.  -Decreasing the incidence of PPH.  . | High risk of bias |
| (5) | Bhavana (2016) | RCT | Term singleton pregnancy delivered by cesarean section. | Subjects with:  -Severe medical and surgical complications.  -History of allergy to tranexamic acid.  -History of thromboembolic disorders.  -Antenatal complications as adherent placenta, placenta-previa or placenta abruption.  -severe preeclampsia.  -Multiple pregnancies.  -Macrosomia.  -Polyhydramnios,  -Fibroid uterus.  -Previous caesarean section. | Tranexamic Acid | over 10 min | 20 ml of normal saline | NA | Injection tranexamic acid showed decreasing blood loss during surgery. | Some concern |
| (6) | Chandak 2015 | RCT | Full-term primiparas or  multiparas with singleton pregnancy being delivered by  lower segment cesarean section. | -Subjects having medical problems including the liver, heart, kidney and brain  -Subjects having blood disorders.  -Subjects having allergy to TA.  -History of severe pre-eclampsia, thromboembolic disorders, multiple pregnancy, abnormal placentation, macrosomia, polyhydramnios and those needing blood transfusion due  to anemia. | TXA | over 5 min | Not given TXA. | NA | Tranexamic acid showed:  -significantly reduction in the quantity of blood loss during and  after the LSCS.  -No complications or side effects were reported. | Some concern |
| (7) | Diop (2020) | RCT | Women who had to deliver vaginally. | Women with history of thrombosis or a clear contraindication for TA such as a known allergy | tranexamic acid | NR | placebo in addition to 800 mcg sublingual misoprostol | NR | Adjunct use of oral TXA with misoprostol in treatment of PPH resulted in similar clinical and acceptability outcomes in comparison with misoprostol alone. | Some concern |
| (8) | Ducloy-Bouthors (2012) | RCT | Patients with post-partum hemorrhage >500 ml. | -Women aged <18 years.  -Absence of informed consent.  -Caesarean section.  -Presence of known hemostatic abnormalities before pregnancy.  -History of thrombosis or epilepsy. | tranexamic acid | (Loading dose 4 g over 1 hour, then infusion of 1 g/hour over 6 hours | nothing | NA | This study showed that TXA reduces blood loss and maternal morbidity in PPH. | High risk of bias |
| (9) | El-Gaber 2018 | RCT | Patients underwent elective caesarean delivery for obstetric indications. | Subjects with:  -Multiple pregnancy,  -macrocosmic baby.  -IUFD.  -Preterm baby.  -Polyhydramnios.  -Presence of uterine fibroids.  -Placenta previa and morbidly adherent placenta.  -Bleeding disorders.  -Presence of any degree of anemia,  -Any medical disorders as liver or heart diseases.  -Anticoagulant therapy.  -History of allergy to TA. | Tranexamic Acid | over 2 minutes | saline | NA | TA administration few minutes prior to elective CS was effective in reducing the incidence and severity of PPH and also decreased additional surgical interventions and the use of additional uterotonic drugs. | Some concern |
| (10) | El-Garhy 2018 | RCT | Full term pregnant with singleton pregnancy who  delivered vaginally. | Women who:  -aged <18 years.  -delivered by CS.  Women with:  -Hemostatic abnormalities.  -History of epilepsy or thrombosis.  -History of medical problems as liver, kidney, heart and brain.  -Allergy to TA.  -Abnormal placentation.  -Antepartum hemorrhage.  -Uterine scar.  -Severe preeclampsia.  -Multiple pregnancy.  -Macrosomia.  -Polyhydramnios.  -severe anemia. | Tranexamic Acid | over 5 minutes | Placebo | NA | Prophylactic administration of TA significantly can reduce blood  loss through and after delivery. | High risk of bias |
| (11) | Fahmy (2021) | RCT | Singleton pregnancy, age from 18 to 39 years old, P1-CS (previous one section after failed consent for trial of labor after CS), term ≥ 37 weeks of gestation, elective CS and spinal anesthesia. | Subjects with:  -Failed spinal anesthesia.  -Multiple pregnancy.  -Grand multipara.  -Placenta previa or abruptio placentae.  -polyhydramnios.  -Fever.  -Rupture of membranes.  -Patients on antiplatelets or anticoagulants.  -Eclampsia or pre-eclampsia,  -History of cardiovascular diseases.  - History of venous thromboembolism, thrombophilia or active thromboembolic disease.  -Allergy to TXA.  -Pre-existing hematuria.  -History of renal insufficiency. | Tranexamic acid | NA | 10 I.U. oxytocin | NA | Prophylactic administration of TA reduced intraoperative and postoperative bleeding in CS and the incidence of PPH. | Some concern |
| (12) | Farahat 2019 | RCT | Clinically free singleton antenatal women, age from 20 to 35 years old. Primary, 2nd or 3rd gravida, more than or equal 38 weeks gestation,  planed for elective CS with normal platelet count range. | Subjects with:  -Allergy to TA.  -With risk factor for PPH. (sever pre-eclampsia, | Tranexamic Acid | over 5 min | 30ml 5% glucose | NA | Results: The mean intraoperative and postpartum blood loss was significantly lower  in the study group than the control group. Conclusion: Preoperative IV tranexamic acid significantly reduced blood loss during  elective CS without any significant adverse effects. | High risk of bias |
| (13) | Gai 2003 | RCT | Term primipara with a singleton pregnancy delivered by CS. | Subjects with:  -Severe medical and surgical complications including the liver, kidney, heart, brain disease and blood disorders. -Allergy to TA. -History of thromboembolic disorders. -Abnormal placenta as placenta-previa or placenta-abruptio. -Pre-eclampsia. -Multiple pregnancies.  -Macrosomia.  -Polyhydramnios. | Tranexamic Acid | over 5 min | nothing | NA | TA statistically reduces PPH. | Some concern |
| (14) | Gobbur 2014 | RCT | Women undergoing lower segment cesarean section. | Subjects with:  -Medical or surgical problem.  -Abnormal placentas as placenta previa or placental abruption.  -Pre-eclampsia.  -Multiple pregnancy. -Macrosomia.  -Polyhydramnios.  -complication with myoma. | tranexamic acid | over 5 min | no intervention | NA | TA significantly reduces the blood loss during and after the CS. | High risk of bias |
| (15) | Goswami (2013) | Double-blind randomized, case controlled prospective trial | ASA grade I and II patients with age more than 18 years and anemic patients with hemoglobin (7-10 g%). | -Subjects belonging to (ASA) physical status III and IV.  -Patients with history of coagulopathy or thromboembolism.  -Patients who had received Acenocoumerol or platelet antiaggregant as Aspirin in the week before surgery.  -Patients having pre-operative plasma creatinine more than 130 μmol/L.  -Patients with history of MI or unstable angina or chronic arteriopathy in the previous 12 months.  - Patients having a history of hepatic or renal impairment.  -Hypersensitivity to TXA. | tranexamic acid | NA | placebo | NA | TXA in doses of 10 mg/kg and 15 mg/kg, was found to be significantly effective in reducing blood loss and also transfusion requirements in anemic patients. | Some concern |
| (16) | Gungorduk (2010) | RCT | Subjects with gestational age more than 38 weeks and they required elective CS. | -Women had risk factors associated with an increased PPH as:  -Anemia (hemoglobin <7 g%).  -Multiple gestation. -Antepartum hemorrhage.  -Abnormal placentation.  -Uterine fibroids.  -Polyhydramnios.  -Emergency CS.  -History of uterine atony and postpartum bleeding. | tranexamic acid | NA | 5% glucose | NA | -This study showed that TA significantly reduced bleeding after CS, additional use of uterotonic agents and the percentage of patients with blood loss >1000 ml. -Also, no increase in the incidence of thromboembolic events was detected. | Low risk of bias |
| (17) | Gungorduk (2013) | RCT | Subjects with gestational age between 34 and 42 weeks, a live fetus with cephalic presentation and expected vaginal birth.  Subjects with risk factors for PPH, such as polyhydramnios, multiple gestation, estimated fetal weight about 4,500 g, grand multiparity, preeclampsia, or previous PPH. | Subjects with:  -Placenta previa or placental abruption. -CS, or any uterine scarring.  -Abnormal placentation  -History of thromboembolic disease. -History of significant disease, including liver, renal disorders and heart disease. | tranexamic acid | NA | 5% glucose | NA | The use of TA reduced postpartum blood loss, and no increase in the incidence of thromboembolic events was detected. | Some concern |
| (18) | Halifa 2021 | RCT | Women who underwent elective or emergency cesarean deliveries. | Women with:  -Severe medical and surgical complications  -Allergy to TXA.  -Known risk factors for PPH. | tranexamic acid | over 10 minutes | placebo | over 10 minutes | TXA reduced blood loss significantly during CS with no adverse events. | Low risk of bias |
| (19) | Hassan (2020) | RCT | Adult pregnant women between 18 to 38 years old, primigravida or multigravida without history of previous CS, at term (37 – 42 weeks), with singleton pregnancies and booked for elective CS. | -Medical and obstetric disorders | oxytocin | over 1 minute | tranexamic acid | over 5 minutes | TA can help against PPH with no considerable side effects. | Some concern |
| (20) | Hasan 2021 | RCT | -Women aged 18 years or more with singleton pregnancy at ≥ 35 weeks of.  Gestation with an intention to vaginal delivery.  -Women with twin pregnancy, grand multiparity, a previous history of PPH, polyhydramnios, a previous history of CS, prolonged labor, suspected macrocosmic fetus or HELLP syndrome. | Women with:  -IUFD.  -Thromboembolic disease’s history.  -Current or previous history of renal and liver disorders, heart disease, seizure or epilepsy.  -Placenta previa or placental abruption. | TA+ oxytocin | over 1 minute | oxytocin | over 1 minute | TA reduced the incidence rates of PPH and the time of placental delivery. | Low risk of bias |
| (21) | Hemapriya 2020 | RCT | Women aged 18 to 35 years undergoing elective or emergency LSCS, beyond 34 weeks of gestation. | Subjects with:  -Anemia (Hb < 10 gm%).  -Hypertension in pregnancy.  -Bleeding diathesis.  -GDM on insulin.  -Polyhydramnios or oligohydramnios.  -Cardiac and chronic liver diseases. | tranexamic acid | NA | no intervention | NA | TA can safely reduce bleeding during emergency and elective LSCS. | High risk of bias |
| (22) | Ibrahim (2019) | RCT | Patients who admitted for elective CS with placenta accreta. | Patients admitted for emergency CS,  with bleeding, preoperative anemia or known to have coagulation disorders. -Pre-eclampsia or GDM. | tranexamic acid | over 10 min | normal saline | NA | TA infusion was effective to reduce intraoperative and postoperative blood loss and also reduce blood products’ transfusion. | Some concern |
| (23) | Ifunanya 2019 | RCT | Pregnant women who had at least one risk factor for PPH  and who were to undergo elective or emergency cesarean delivery. | Subjects with:  -History of renal, cardiac and liver diseases.  -Bleeding disorders.  -History of thrombogenic  episodes or anticoagulant use.  -Allergy to TA. | Tranexamic Acid | over 5 min | normal saline | over 5 min | Intravenous TA given before skin incision at CS reduced incidence of primary PPH and the need for additional uterotonics. | Some concern |
| (24) | Igboke 2022 | RCT | Subjects with spontaneous labor, planned vaginal delivery, singleton, term pregnancy and cephalic presentation. Parturient who have no contraindications to the use  of tranexamic acid. | Women with:  -History of thromboembolism, sickle cell disease, bleeding disorders, autoimmune diseases, liver pathology, renal disease, known cardiovascular disorders.  -Multiple pregnancy, IUFD.  -Previous uterine surgeries.  -Patients with chronic HTN.  -Preeclampsia. -Antepartum hemorrhage or ruptured uterus.  -Varicose veins.  -History of epilepsy or seizures. | tranexamic acid | over 30-60 seconds | water for injection | over 30-60 seconds | Intravenous TA reduced blood loss following **VD** and the need for additional uterotonics. | High risk of bias |
| (25) | Ismail (2017) | RCT | Pregnant women (37-42 weeks), with a live fetus and cephalic presentation who were passed spontaneously in labor and expected to normal vaginal delivery. | -Women with risk factors for PPH as over distended uterus by (polyhydramnios, multiple gestations or macrocosmic baby).  -Grand multipara.  -Women with hypertensive disorders. -Previous history of PPH.  -History of scarred uterus. | tranexamic acid | over a 5-minute period | 5% glucose | over a 5-minute period | The use of intravenous TA after VD reduces postpartum hemorrhage. | Low risk of bias |
| (26) | Jafarbegloo 2017 | RCT | Pregnant women who were to undergo elective cesarean delivery, aged 18–35 years with a singleton pregnancy at (38-42) weeks' gestation and Blood pressure less than 140/90 mmHg. | Pregnancy complications as  -pre-eclampsia.  -polyhydramnios.  -macrosomia.  -preterm labor.  -multiple pregnancies.  -placenta previa or abruptio placenta.  -abnormal placenta.  -thrombophilia, coagulopathy, anemia, cardiovascular, liver, renal disorders.  -allergy to TA. | Tranexamic Acid | over 10 min | distilled water | over 10 min | Pre-operative prescription of TA was not associated with improvement of post-operative hematocrit and hemoglobin. | Some concern |
| (27) | Kafayat (2018) | RCT | Primigravida and multi-parous women, at term pregnancy and undergoing elective CS. | Women with abnormal coagulation profile, preeclampsia, , anemia, abnormal placentation, multiple pregnancy or macrosomia. | tranexamic acid | over 5min | nothing | NA | Use of TA reduced blood loss, but didn’t not reduce Hb in women giving the birth by CS. | High risk of bias |
| (28) | Kamel 2018 | RCT | Women with parity not more than two, singleton pregnancy. delivery by elective LSCS. aged between 18 and 37 years old and gestational age of 37 to 42 weeks of pregnancy. | Subjects with:  -Twin pregnancy.  -Vaginal delivery or urgent C S.  -History of thromboembolic  disorders.  -Tendency for increased bleeding  as abnormal placentation, polyhydramnios, multiple pregnancy, previous two or more  CS. | tranexamic acid and oxytocin | over 15 min | no intervention | NA | The use of TA prior to CS is significantly effective in reducing blood loss with no observed neonatal or maternal side effects. | High risk of bias |
| (29) | Kashanian (2021) | RCT | Multiparous women with singleton pregnancy, gestational age between 37 and 42 weeks with normal blood pressure (<140/90 mmHg). | Subjects with:  -History of coagulopathy.  -Pre-eclampsia.  -Placental abruption,  -Hypersensitivity to TXA.  -History of cardiac, renal, hepatic and neurologic disorders.  -BMI > 30 and episiotomy. | tranexamic acid | NA | distilled water | NA | TA can reduce the amount of bleeding after VD. Also, it can decrease the need for additional uterotonic agents | Low risk of bias |
| (30) | Lakshmi (2016) | RCT | Pregnant women aged between 19 and 34 years with gestational age of 37 to 42 weeks. | Subjects with:  -Medical problems as GDM, hypertension, renal disease or heart disease.  -Coagulation disorders.  -Allergy to TXA.  -History of thromboembolic disorders.  -Abnormal placentation, polyhydramnios, multiple pregnancy or previous two or more CS. | tranexamic acid | NA | nothing | NA | TA reduced the amount of blood loss significantly during LSCS. | High risk of bias |
| (31) | Maged (2015) | RCT | Women with full-term singleton pregnancies who were scheduled to undergo an elective LSCS. | -Subjects with anemia, maternal medical disorders (e.g., renal, cardiac and hepatic diseases), known allergy to TA or history of thromboembolic events.  -Patients with an increased  risk of obstetric hemorrhage. | tranexamic acid | NA | glucose | NA | Preoperative administration of TA reduced safely blood loss during elective LSCS. | High risk of bias |
| (32) | Matloob (2021) | RCT | Patients aged 17 to 45 years with term pregnancy i.e., 37 weeks and 6 days till 41 weeks and 6 days, assessed by ultrasound and date of LMP. Vertex presentation of fetus, assessed by ultrasound and expected spontaneous delivery. | Subjects with retained products of placenta detected by ultrasound. | tranexamic acid | NA | misoprostol | NA | Administration of TA reduced incidence of PPH. | High risk of bias |
| (33) | Mbah 2021 | RCT | Women of reproductive  age, singleton, and term pregnancy. | Subjects with:  -obstructed labor  -prolonged labor.  -polyhydramnios.  -IUFD.  -previous history of PPH.  -history of thromboembolism.  -abnormal placentation.  -bleeding disorders.  -antepartum hemorrhage.  -allergy to TA. | tranexamic acid | over 5 min | water for injection | over 5 min | Intravenous TA given previous to skin incision at CS reduced significantly perioperative blood loss, the incidence of primary PPH and the need for blood transfusion. | Some concern |
| (34) | Milani (2019) | RCT | Multipara or primipara singleton pregnant women at (37-40) weeks of gestation and ASA class I and II. | Subjects with:  -Medical diseases including kidney, liver, heart and blood disorders; -History of thromboembolic disease, severe preeclampsia, abnormal placenta, macrosomia, multiple pregnancies, and polyhydramnios. -Anemia. -Rheumatic diseases or obesity. -Varicose veins or history of DVT. | tranexamic acid | NA | distilled water | NA | 1g of TXA could reduce the volume of PPH significantly. Though, it did not cause differences in hemodynamic and Hb levels. | Some concern |
| (35) | Mirghafourvand (2015) | RCT | Women aged 18–35 years with a singleton pregnancy with a cephalic presentation, between (38- 42) weeks’ gestation, normal blood pressure (< 140/90 mmHg) and intended to deliver vaginally. | -Grand multipara.  -Prolonged labor  -Previous CS or uterine surgery.  -Uterine myoma.  -History of hepatic, cardiac, renal and neurologic disorders.  -History of blood disorders or  thromboembolic disease.  -History of pre-eclampsia, diabetes, PPH in previous pregnancies. | tranexamic acid | NA | placebo | NA | TA is found to reduce PPH significantly. | Low risk of bias |
| (36) | Movafegh (2011) | RCT | Women aged 20–40 years with a singleton pregnancy between 38 weeks+5 days and 40 weeks’ gestation, who were categorized as class 1 (normally healthy) according to ASA and were scheduled to undergo cesarean delivery. | -Previous history of CS.  -Polyhydramnios. -Macrosomia.  -Pre-eclampsia.  -Abnormal placenta.  -Thrombophilia, anemia, or coagulopathy.  -Cardiovascular, liver or renal disorders. | tranexamic acid | over 10 minutes | normal saline | over 10 minutes | Administration of 10 mg/kg of intravenous TA before skin incision in women undergoing CS reduces intraoperative and postoperative blood loss and as well oxytocin use | Some concern |
| (37) | Naeiji (2021) | RCT | Singleton pregnant women at term with indication of elective CS. | Patients with:  -Known allergy to TXA.  -History of coagulative or thromboembolic disorders.  -History of cardiac, renal or hepatic impairment.  -Pre-eclampsia.  -Placental disorders or polyhydramnios. | tranexamic acid | NA | distilled water | NA | Prophylactic use of intravenous TA decreases safely intraoperative and post-operative blood loss in women undergoing elective CS. | Low risk of bias |
| (38) | Nargis (2018) | RCT | Subjects aged > 18 years, term >35 weeks of gestation, singleton pregnancy, elective CS. | Subjects with:  -History of venous or arterial thrombosis, epilepsy or seizure.  -History of any known cardiovascular, liver or renal disorders, sickle cell disease, autoimmune disease or severe hemorrhagic disease.  -Placenta previa or abruptio placenta. -Eclampsia or HELLP syndrome. -Multiple pregnancy.  -IUFD | tranexamic acid | NA | sterile distilled water | NA | Tranexamic acid reduced effectively blood loss in patients undergoing LSCS. | Some concern |
| (39) | Obi 2019 | RCT | Parturient with singleton pregnancy at (37-42)  weeks’ gestational age, admitted for elective CS. | Women with:  -Known allergy to TA.  -History of bleeding disorders or thromboembolism.  -Medical disorders in pregnancy; liver pathology, renal disease or hypertensive disorders in pregnancy.  - Antepartum hemorrhage. | tranexamic acid | 1 ml/min over 10 min | water for injection | 1 ml/min over 10 min | Intravenous TA reduced blood loss significantly at elective CS. | Low risk of bias |
| (40) | Oseni (2021) | RCT | Patients in the age group more than 18 years to less than 40 years; of low parity, pregnant with singleton, live fetus at gestational age between (37 -42) weeks; who were to have primary emergency CS under spinal anesthesia. | Patients with:  -Known allergy to TA.  -Bleeding disorders.  -Antepartum hemorrhage.  -Patients with major hepatic, cardiac, renal or respiratory disorders. | tranexamic acid | NA | normal saline | NA | TA caused significant reduction in the blood loss during emergency CS and also a significant reduction in PPH and postpartum anemia. | Some concern |
| (41) | Ramani 2014 | RCT | Patient undergoing emergency CS. | Women with:  -Liver disorder, heart diseases, renal problem.  -Allergy to TA.  -Multifetal gestation.  -Eclampsia and severe pre-eclampsia. | tranexamic acid | over 10 min | no intervention | NA | Tranexamic acid is effective and safe in preventing PPH after CS. | High risk of bias |
| (42) | Ray (2016) | RCT | Stable singleton mothers (aged 20–40 years) at  term planned for elective CS. | Women with:  -Pregnancy complications as pre-eclampsia, multiple pregnancy, polyhydramnios, preterm labor, macrosomia.  -Placenta previa or abruptio placentae.  -Blood dyscrasias, thromboembolic disorders, coagulation disorders or severe anemia. -Allergy to TA. | tranexamic acid | NA | IV placebo i.e., 30 ml of 5 % Dextrose solution | NA | Antenatal administration of intravenous TA 20 min before spinal anesthesia reduced significantly the amount of blood loss during and after LSCS. | High risk of bias |
| (43) | Roy (2016) | RCT | Primi and second gravida with more than 38 weeks of gestation and spontaneous or induced labor. | Subjects with: -Twin pregnancy. - Polyhydramnios. - Macrosomia. - Previous history PPH. - Fibroid complicating pregnancy. - Placenta previa or abruptio placenta. - PROM.  -Prolonged and obstructed labor. | tranexamic acid | NA | normal saline (5 ml) | NA | TA injection appears to effectively reduce the blood loss and maternal morbidities during normal labor. | Some concern |
| (44) | Sekhavat (2009) | Prospective, randomized, case-controlled clinical trial | Patients with indications for CS as complicated pregnancy, abnormal pelvis, abnormal presentation, fetal distress, old primipara and refusal of VD. | Women with:  -Severe medical and surgical disorders.  -Blood disorders and anemia.  -Allergy to TA. -History of thromboembolic disorders.  -Abnormal placenta as placenta-previa or placenta-abruption. -Severe preeclampsia.  -Multiple pregnancies, polyhydramnios, macrosomia and those requiring blood transfusion. | tranexamic acid | over 5 min | 5% dextrose | NA | TA can safely reduce bleeding after CS without any side effects or complications. | High risk of bias |
| (45) | Sentilhes (2018) | RCT | Women at age group of 18 years or older who had a singleton pregnancy at 35 weeks, and planning to undergo vaginal delivery. | Women with:  -Risk of venous or arterial thrombosis.  -Risk bleeding. -History of epilepsy or seizure. | tranexamic acid | NA | placebo | NA | The use of TA did not result in a rate of PPH of at least 500 ml which was significantly lower than the rate with placebo. | Low risk of bias |
| (46) | Sentilhes 2021 | RCT | Women undergoing CS before or during labor at 34 or more gestational age. | Women with:  -known or possible risk of venous or arterial thrombosis  -History of bleeding.  -History of epilepsy or seizure.  -Prenatal hemoglobin level of 9 g per  deciliter or lower., | tranexamic acid | over a pe- riod of 30 to 60 seconds | normal saline | over a pe- riod of 30 to 60 seconds | TA treatment reduced significantly incidence of calculated estimated blood loss but it did not result in a reduction incidence of hemorrhage related to secondary clinical outcomes. | Low risk of bias |
| (47) | Sentürk (2013) | RCT | Women undergoing elective or urgent CS. | Patients with:  -High BMI.  -Venous thromboembolism.  -Uterine myoma.  -Active liver or kidney diseases.  -Polyhydramnios and macrosomia.  -Allergies to TA. | tranexamic acid | NA | 5 % dextrose | NA | Use of TA reduced safely bleeding in urgent or elective CS. | Some concern |
| (48) | Shah 2018 | RCT | Pregnant women, with singleton pregnancy of 37 weeks or more of gestation, who were planned for elective CS. | Women with:  -History of medical disorders (liver, renal disorders, heart  disease, hypertension, diabetes mellitus or a known coagulopathy).  -History of sensitivity or contraindication to TA. | tranexamic acid | 10 mins before skin incision | normal saline | 10 mins before skin incision | Preoperative use of TA is associated with reduction in blood loss during and after elective  CS. | High risk of bias |
| (49) | Shahid 2013 | RCT | Primipara or multiparas with Full term, singleton pregnancy being delivered by LSCS. | Subjects with:  -Medical problems including the liver, heart, brain, kidney and having blood disorders.  -Allergy to TA.  -History of thromboembolic disorders.  -Abnormal placentation, multiple pregnancy, macrosomia, severe preeclampsia, polyhydramnios and those needing blood transfusion. | tranexamic acid | 10 mins before skin incision | distilled water | 10 mins before skin incision | TA reduced the amount of blood loss significantly during the LSCS, but not after the CS. | Some concern |
| (50) | Sharma 2011 | RCT | Subjects at age group between (20 – 30) years, with full term primipara or multipara with single pregnancy being delivered by CS. | Subjects with:  -Medical disorders and blood disorders.  -History of thromboembolic disorders.  -Hypersensitivity to TA.  -Multiple pregnancy or polyhydramnios.  -Anemia requiring blood transfusion. | tranexamic acid | over 5 min | nothing | NA | TA reduced the amount of blood loss significantly during and after the LSCS. | High risk of bias |
| (51) | Singh 2014 | RCT | Primipara or multipara delivered by CS without any risk factors. | Patient with:  -Hemorrhagic disorder.  -Placenta previa.  -Polyhydramnios, twin pregnancy, PIH, anemia. | tranexamic acid | over 5 min | nothing | NA | TA reduced significantly the  amount of blood loss during and after LSCS. | High risk of bias |
| (52) | Soliman (2021) | NA | Pregnant females at age group between 18 and 35 years, with singleton alive fetus and went through elective CS. | Subjects who did not meet the inclusion criteria. | tranexamic acid | NA | nothing | NA | Tranexamic acid administration before elective cesarean section was effective in decreasing intraoperative and postoperative bleeding. And in turn reduces the incidence of PPH with no immediate maternal or neonatal side effects. | Some concern |
| (53) | Sujata (2016) | RCT | Women with at least one risk factor for PPH and were to undergo emergency or elective CS.  The risk factors considered were:  - PIH  - Use of oxytocin augmentation for at least 4 h. - More than two previous CS. - Chorioamnionitis. - General anesthesia.  - Placenta previa.  - Polyhydramnios.  - Fibroids.  - Multiparity  - Multiple pregnancy.  - Cholestasis.  - Macrosomia. - Genital-tract injury. | -Patients who had emergency CS.  -History of hemodynamic instability, ischemic cardiac disease or bleeding disorders.  -Allergy to TA.  -History of thrombogenic episodes or anticoagulant use. | tranexamic acid | NA | nothing | NA | Intravenous TA administration before skin incision, reduced the requirement for additional uterotonics significantly among women at increased risk for PPH. | Some concern |
| (54) | Sujita (2018) | RCT | Women at age group more than 18 years, GA between 36 and 42 weeks, with singleton, a live fetus and cephalic presentation in the active phase of labor. | Subjects with:  -Multiple pregnancy.  -IUFD.  -Vacuum extraction or forceps extraction.  -Placenta previa, placenta accreta or placental abruption.  -Previous CS or any uterine scar.  -History of heart disease, thromboembolic disease, liver and renal disorders | tranexamic acid | NA | placebo | NA | The addition of TA in vaginal delivery did not reduce the amount of postpartum blood loss in the first two hours in comparison to prophylaxis oxytocin only. | Some concern |
| (55) | Tabatabaie (2021) | RCT | Pregnant women at age group between (18-40) years with a GA of 37-42 weeks and the candidates for LSCS. | Subjects with:  -Multiple pregnancies, macrosomia, placental abnormalities, polyhydramnios or preeclampsia.  -Previous CS or intra-abdominal surgery.  -Cardiovascular disease, liver disease, renal disease, brain problems, coagulopathy, blood disorders, thrombophilia, severe anemia (Hb less than 8 mg/dL) or thromboembolic disorders  -Allergy to TA.  -BMI>30.  -General anesthesia and all spinal anesthesia contraindications. | tranexamic acid | NA | group B: misoprostol, group c: placebo | NA | Both TA and Misoprostol reduced bleeding volume during LSCS. | High risk of bias |
| (56) | Taj 2014 | RCT | Subjects with singleton gestation who planned to have CS at 37 weeks. | NR | tranexamic acid | over 10 min | undefined placebo | NA | Administration of TA was associated with reduction in blood loss during and after CS. | High risk of bias |
| (57) | Torky (2021) | RCT | Women undergoing elective CS. | Subjects with:  -Hypersensitivity or contraindications to TA.  -Bleeding or coagulation disorders.  -Placenta previa. | tranexamic acid | NA | group b: Etamsylate,  group c: normal saline | NA | Etamsylate is an effective second-line therapy, after TA, in reducing blood loss during an elective CS with low risk of side effects. | Some concern |
| (58) | Xu (2013) | Randomized, double-blind, case-controlled | Patients who had blood loss more than 500 ml after delivery. | Patients with:  -Age was less than 18 years.  -Severe medical and surgical complications.  -Allergy to TA.  -Multiple pregnancies, polyhydramnios or macrosomia.  -Hemostatic abnormalities. | tranexamic acid | over 10 and 20 min | normal saline | over 10 and 20 min | Administration of 10 mg/kg of TA before beginning spinal anesthesia in women undergoing CS is effective in reducing postoperative blood loss. | Some concern |
| (59) | Yehia 2014 | RCT | Women attending for elective CS. | Women with:  -Bleeding tendency.  -Risk of thromboembolism.  -Allergy to TA.  -Ante-partum hemorrhage or abnormal site of the placenta. | tranexamic acid and oxytocin | over 2 min | oxytocin alone | NA | Tranexamic acid can reduce safely blood loss during CS and improve post-operative hemoglobin and hematocrit. | Some concern |

TA; tranexamic acid, RCT; randomized control trial, PPH; postpartum hemorrhage, CS; cesarean section, LSCS; lower segment cesarean section, PIH; pregnancy induced hypertension, VD; vaginal delivery, BMI; body mass index, GA; gestational age, PROM; premature rupture of membranes, LMP; last menstrual period, IUFD; intrauterine fetal death, GDM; gestational diabetes mellitus, DVT; deep vein thrombosis, MI; myocardial infarction, BUAL; bilateral uterine artery ligation. HELP; hemolysis, elevated

liver enzymes, and low platelets.

**Supplemental Data Legend:**

**Supplemental Digital Content, Figure 1***. Publication Bias*

**Supplemental Digital Content, Table 1***. Summary of the included studies*

**Supplemental Digital Content, Table 2***. Baseline characteristics of the included studies*

**Supplemental Digital Content, Table 3***. Assessment risk of bias of the included studies*

References

1. Abdel-Aleem H, Alhusaini TK, Abdel-Aleem MA, Menoufy M, Gülmezoglu AM. Effectiveness of tranexamic acid on blood loss in patients undergoing elective cesarean section: Randomized clinical trial. Journal of Maternal-Fetal and Neonatal Medicine. 2013;26:1705-9.

2. Ahmed MR, Sayed Ahmed WA, Madny EH, Arafa AM, Said MM. Efficacy of tranexamic acid in decreasing blood loss in elective caesarean delivery. Journal of Maternal-Fetal and Neonatal Medicine. 2015;28:1014-8.

3. Ali MM, El-Bromboly WH, Elnagar WM, Hashem MFA. Prevention of postpartum hemorrhage after vaginal delivery using tranexamic acid. Egyptian Journal of Hospital Medicine. 2021;85:2937-40.

4. Nivedhana AP, Indu NR, Jalakandan B. Does prophylactic tranexamic acid reduce blood loss in Indian women following vaginal delivery? International Journal of Reproduction, Contraception, Obstetrics and Gynecology. 2021;10:497+.

5. G. B, MV A, Mittal S. Efficacy of prophylactic tranexamic acid in reducing blood loss during and after caesarean section. International Journal of Reproduction, Contraception, Obstetrics and Gynecology. 2016;5:2011-6.

6. Chandak AV, Gupta I. Efficacy of Tranexamic Acid in Decreasing Blood Loss during and after Cesarean Section: A Randomized Case Controlled Prospective Study. International Journal of Science and Research (IJSR) ISSN. 2015;6.

7. Diop A, Abbas D, Ngoc NTN, Martin R, Razafi A, Tuyet HTD, et al. A double-blind, randomized controlled trial to explore oral tranexamic acid as adjunct for the treatment for postpartum hemorrhage. Reproductive Health. 2020;17:1-7.

8. Ducloy-Bouthors AS, Jude B, Duhamel A, Broisin F, Huissoud C, Keita-Meyer H, et al. High-dose tranexamic acid reduces blood loss in postpartum haemorrhage. Critical Care. 2011;15:1-10.

9. Abd El-Gaber AE-N, Ahmed HH, Khodry MM, Abbas AM. Effect of tranexamic acid in prevention of postpartum hemorrhage in elective caesarean delivery: a randomized controlled study. International Journal of Reproduction, Contraception, Obstetrics and Gynecology. 2018;8:1.

10. El-Garhy ET, Mohamed AH, Elshahat A, Abu Elmagd I, Hamed MAA. Tranexamic Acid for Prevention of Postpartum Hemorrhage after Vaginal Delivery. The Egyptian Journal of Hospital Medicine. 2018;73(2):6157-64.

11. Fahmy NG, Eskandar FSL, Khalil WAMA, Sobhy MII, Amin AMAA. Assessment the role of tranexamic acid in prevention of postpartum hemorrhage. Ain-Shams Journal of Anesthesiology. 2021;13.

12. Farahat  MA. Role of intravenous tranexamic acid on cesarean blood loss: a prospective randomized study. Women's Health. 2019;8:226-30.

13. Gai MY, Wu LF, Su QF, Tatsumoto K. Clinical observation of blood loss reduced by tranexamic acid during and after caesarian section: A multi-center, randomized trial. European Journal of Obstetrics and Gynecology and Reproductive Biology. 2004;112:154-7.

14. Gobbur V, Shiragur S, Jhanwar U, Tehalia M. Efficacy of tranexamic acid in reducing blood loss during lower segment caesarean section. International Journal of Reproduction, Contraception, Obstetrics and Gynecology. 2014;3:414-7.

15. Goswami U, Sarangi S, Gupta S, Babbar S. Comparative evaluation of two doses of tranexamic acid used prophylactically in anemic parturients for lower segment cesarean section: A double-blind randomized case control prospective trial. Saudi Journal of Anaesthesia. 2013;7:427-31.

16. Gungorduk K, Yildirim G, Asicioǧlu O, Gungorduk OC, Sudolmus S, Ark C. Efficacy of intravenous tranexamic acid in reducing blood loss after elective cesarean section: A prospective, randomized, double-blind, placebo-controlled study. American Journal of Perinatology. 2011;28:233-9.

17. Gungorduk K, Asicioǧlu O, Yildirim G, Ark C, Tekirdaǧ A, Besimoglu B. Can intravenous injection of tranexamic acid be used in routine practice with active management of the third stage of labor in vaginal delivery? A randomized controlled study. American Journal of Perinatology. 2013;30:407-13.

18. Halifa I, Olusesan Oluwasola T, Fawole B, Oladokun A. Intravenous tranexamic acid for reducing blood loss during cesarean delivery: A double-blind, randomized-controlled trial. New Nigerian Journal of Clinical Research. 2021;10:40.

19. Hassan NE, Elghareeb NAM, Zaki FM. Efficacy of oxytocin infusion versus tranexamic acid infusion in controlling blood loss during elective lower segment caesarean section. Egyptian Journal of Hospital Medicine. 2020;81:1822-7.

20. Hasan CS, Alalaf SK, Khoshnaw SA. Tranexamic Acid Administration for the Prevention of Blood Loss After Vaginal Delivery in a High-Risk Pregnancy: A Double-blind Randomized Controlled Trial. 2021.

21. Hemapriya L, More G, Kumar A. Efficacy of Tranexamic Acid in Reducing Blood Loss in Lower Segment Cesearean Section: A Randomised Controlled Study. The Journal of Obstetrics and Gynecology of India. 2020;70(6):479-84.

22. Ibrahim TH. Efficacy of tranexamic acid in reducing blood loss, blood and blood products requirements in Cesarian sections for patients with placenta accreta. Ain-Shams Journal of Anesthesiology. 2019;11:2-7.

23. Ifunanya NJ, Chukwu IC, Nobert OC, Blessing O, Chibuzor UD-P, Uchenna OV. Tranexamic Acid versus Placebo for Prevention of Primary Postpartum Haemorrhage among High Risk Women Undergoing Caesarean Section in Abakaliki: A Randomized Controlled Trial. Open Journal of Obstetrics and Gynecology. 2019;09:914-22.

24. Igboke FN, Obi VO, Dimejesi BI, Lawani LO. Tranexamic acid for reducing blood loss following vaginal delivery: a double-blind randomized controlled trial. BMC Pregnancy Childbirth. 2022;22(1):178.

25. Ismail A, Abbas A, Shahat M, Ali K. Evaluation of Subendometrial and Intramyometrial Blood Flow after Intravenous Tranexamic Acid for Prevention of Postpartum Hemorrhage in Vaginal Delivery: A Randomized Controlled Study. Journal of Gynecological Research and Obstetrics. 2017;3:046-50.

26. Jafarbegloo E, Faridnyia F, Nejad ASMH. The Effect of Intravenous Tranexamic Acid on Hemoglobin and Hematocrit Levels After Cesarean Delivery: a Randomized Controlled Clinical Trial. 2021.

27. Kafayat H, Janjua M, Naheed I, Iqbal T. To assess the prophylactic role of tranexamic acid in reducing blood loss during and after two hours of caesarean section. Pakistan Journal of Medical and Health Sciences. 2018;12:1662-5.

28. Kamel HEH, Farhan AM, Abou Senna HF, Khedr MA, Albhairy AA. Role of Prophylactic Tranexamic Acid in Reducing Blood loss during Elective Caesarean section in Rural Area. The Egyptian Journal of Hospital Medicine. 2018;73:6886-96.

29. Kashanian M, Dadkhah F, Tabatabaei N, Sheikhansari N. Effects of tranexamic acid on the amount of bleeding following vaginal delivery and its adverse effects: a double-blind placebo controlled randomized clinical trial. Journal of Maternal-Fetal and Neonatal Medicine. 2021;0:1-5.

30. Dhivya Lakshmi SJ, Abraham R. Role of prophylactic tranexamic acid in reducing blood loss during elective caesarean section: A randomized controlled study. Journal of Clinical and Diagnostic Research. 2016;10:OC17-OC21.

31. Maged AM, Helal OM, Elsherbini MM, Eid MM, Elkomy RO, Dahab S, et al. A randomized placebo-controlled trial of preoperative tranexamic acid among women undergoing elective cesarean delivery. International Journal of Gynecology and Obstetrics. 2015;131:265-8.

32. Matloob M, Hyder Syed Z, Qasim R, Najeeb W. Comparison of Misoprostol With Tranexamic Acid in Preventing Post-Partum Hemorrhage. Pakistan Journal of Medical and Health Sciences. 2021;15(5):914-6.

33. Mbah K, Omietimi J, Oyeyemi N, Abasi I, Allagoa D, Oriji P, et al. The Efficacy of Prophylactic Tranexamic Acid in Reducing Perioperative Blood Loss During Caesarean Section: A Randomized, Double Blind Control Trial. Journal of Gynecology and Womens Health. 2021;21.

34. Milani F, Haryalchi K, Sharami SH, Atrkarroshan Z, Farzadi S. Prophylactic effect of tranexamic acid on hemorrhage during and after the cesarean section. International Journal of Women's Health and Reproduction Sciences. 2019;7:74-8.

35. Mirghafourvand M, Alizadeh Charandabi SM, Abasalizadeh F, Shirdel M. The effect of intravenous tranexamic acid on hemoglobin and hematocrit levels after vaginal delivery: A randomized controlled trial. Iranian Journal of Obstetrics, Gynecology and Infertility. 2013;16:1-8.

36. Movafegh A, Eslamian L, Dorabadi A. Effect of intravenous tranexamic acid administration on blood loss during and after cesarean delivery. International Journal of Gynecology and Obstetrics. 2011;115:224-6.

37. Naeiji Z, Delshadiyan N, Saleh S, Moridi A, Rahmati N, Fathi M. Prophylactic use of tranexamic acid for decreasing the blood loss in elective cesarean section: A placebo-controlled randomized clinical trial. Journal of Gynecology Obstetrics and Human Reproduction. 2021;50:101973.

38. Nargis N, Dewan F. Prophylactic use of tranexamic acid during caesarean section in preventing postpartum haemorrhage-a prospective randomised double blind placebo controlled study. Bangladesh Journal of Obstetrics and Gynecology. 2018;33:125-30.

39. Obi CN. Efficacy of intravenous tranexamic acid at reducing blood loss during elective caesarean section in Abakaliki: A double blind randomized placebo controlled trial. African Journal of Medical and Health Sciences. 2019;18:10-7.

40. Oseni RO, Zakari M, Adamou N, Umar UA. Effectiveness of preoperative tranexamic acid in reducing blood loss during caesarean section at Aminu Kano teaching hospital, Kano: A randomized controlled trial. Pan African Medical Journal. 2021;39.

41. Ramani B, Nayak L. Intravenous 1 gram tranexamic acid for prevention of blood loss and blood transfusion during caesarean section: a randomized case control study. International Journal of Reproduction, Contraception, Obstetrics and Gynecology. 2014;3:366-9.

42. Ray I, Bhattacharya R, Chakraborty S, Bagchi C, Mukhopadhyay S. Role of Intravenous Tranexamic Acid on Caesarean Blood Loss: A Prospective Randomised Study. Journal of Obstetrics and Gynecology of India. 2016;66:347-52.

43. Roy P, Sujatha MS, Bhandiwad A, Biswas B. Role of Tranexamic Acid in Reducing Blood Loss in Vaginal Delivery. Journal of Obstetrics and Gynecology of India. 2016;66:246-50.

44. Sekhavat L, Tabatabaii A, Dalili M, Farajkhoda T, Tafti AD. Efficacy of tranexamic acid in reducing blood loss after cesarean section. Journal of Maternal-Fetal and Neonatal Medicine. 2009;22:72-5.

45. Sentilhes L, Sénat MV, Le Lous M, Winer N, Rozenberg P, Kayem G, et al. Tranexamic Acid for the Prevention of Blood Loss after Cesarean Delivery. New England Journal of Medicine. 2021;384:1623-34.

46. Sentilhes L, Winer N, Azria E, Sénat M-V, Le Ray C, Vardon D, et al. Tranexamic Acid for the Prevention of Blood Loss after Vaginal Delivery. New England Journal of Medicine. 2018;379(8):731-42.

47. Sentürk MB, Cakmak Y, Yildiz G, Yildiz P. Tranexamic acid for cesarean section: A double-blind, placebo-controlled, randomized clinical trial. Archives of Gynecology and Obstetrics. 2013;287:641-5.

48. Shah P, Agrawal A, Chhetri S, Rijal P, Bhatta NK. Tranexamic acid in prevention of postpartum hemorrhage in elective cesarean section. International Journal of Reproduction, Contraception, Obstetrics and Gynecology. 2019;8:372.

49. Shahid A, Khan A. Tranexamic acid in decreasing blood loss during and after caesarean section. J Coll Physicians Surg Pak. 2013;23(7):459-62.

50. Sharma R, Najam R, Misra MK. Efficacy of Tranexamic Acid in Decreasing Blood Loss During and After Cesarean Section. Biomedical and Pharmacology Journal. 2011;4(1):231-5.

51. Singh T, Burute SB, Deshpande HG, Jethani S, Ratwani K. Efficacy of Tranexamic Acid in Decreasing Blood Loss During and After Caesarean Section: a Randomized Case Control Prospective Study. Journal of Evolution of Medical and Dental Sciences. 2014;3:2780-8.

52. Soliman AA, Mahmoud SA, Dawood RM, Fayed AA, Fathey AA. Prophylactic use of tranexamic acid in reducing blood loss during elective cesarean section. Egyptian Journal of Hospital Medicine. 2021;82:6-10.

53. Sujata N, Tobin R, Kaur R, Aneja A, Khanna M, Hanjoora VM. Randomized controlled trial of tranexamic acid among parturients at increased risk for postpartum hemorrhage undergoing cesarean delivery. International Journal of Gynecology and Obstetrics. 2016;133:312-5.

54. Sujita A, Songthamwat S, Songthamwat M. Effectiveness of tranexamic acid for reducing postpartum blood loss in the first two hours after vaginal delivery: A randomised controlled trial. Journal of Clinical and Diagnostic Research. 2018;12:QC01-QC4.

55. Tabatabaie SS, Alavi A, Bazaz M. Comparison of the effect of tranexamic acid and misoprostol on blood loss during and after cesarean section: A randomized clinical trial. Razavi International Journal of Medicine. 2021;9:e811.

56. Efficacy of Tranexamic acid in reducing blood loss during and after Cesarean section, (2014).

57. Torky H, El-Desouky ES, Abo-Elmagd I, Mohamed A, Abdalhamid A, El-Shahat A, et al. Pre-operative tranexemic acid vs. Etamsylate in reducing blood loss during elective cesarean section: Randomized controlled trial. Journal of Perinatal Medicine. 2021;49:353-6.

58. Xu J, Gao W, Ju Y. Tranexamic acid for the prevention of postpartum hemorrhage after cesarean section: A double-blind randomization trial. Archives of Gynecology and Obstetrics. 2013;287:463-8.

59. Yehia AH, Koleib MH, Abdelazim IA, Atik A. Tranexamic acid reduces blood loss during and after cesarean section: A double blinded, randomized, controlled trial. Asian Pacific Journal of Reproduction. 2014;3:53-6.
