# Supplementary material for: Efficacy and safety of tranexamic acid in prevention of postpartum hemorrhage: a systematic review and meta-analysis of 18,649 patients
Source: BMC Pregnancy Childbirth. 2023 Nov 24;23:817. doi: 10.1186/s12884-023-06100-8 (PMC10668444; doi:10.1186/s12884-023-06100-8)
Supplement: Supplementary file 3 — Additional file 3: Supplementary Table 3. Assessment risk of bias of the included studies. [file 12884_2023_6100_MOESM3_ESM.docx]

**Supplementary Table 3:** Assessment risk of bias of the included studies

| Ref. | Studies | Randomization process | Deviations from intended interventions | Bias in measurement of the outcome | Bias due to missing outcome data | Bias in selection of the reported result | Other bias | overall judgment |
| --- | --- | --- | --- | --- | --- | --- | --- | --- |
| (1) | Abdel-Aleem 2013 | Low | Low | Low | Low | Low | Low | low |
| (2) | Ahmed 2014 | Low | Low | Low | Low | Unclear | Low | some concern |
| (3) | Ali 2021 | Low | Low | Low | Unclear | High | Low | high |
| (4) | Arthi 2021 | Unclear | Low | Low | High | Unclear | Low | high |
| (5) | Bhavana 2016 | Unclear | Low | Low | Low | Unclear | Low | some concern |
| (6) | Chandak 2015 | Low | Unclear | Low | Low | Unclear | Low | some concern |
| (7) | Diop 2020 | Unclear | Low | Low | Low | Low | Low | some concern |
| (8) | Ducloy-Bouthors 2011 | Low | High | Low | Low | Unclear | Low | high |
| (9) | El-Gaber 2018 | Low | Low | Low | Low | Unclear | Low | some concern |
| (10) | El-Garhy 2018 | High | Low | Low | Low | Unclear | Low | high |
| (11) | Fahmy 2021 | Low | Low | Low | Low | Unclear | Low | some concern |
| (12) | Farahat 2019 | Unclear | Unclear | Low | Low | Unclear | Low | high |
| (13) | Gai 2003 | Low | Low | Low | Unclear | Unclear | Low | some concern |
| (14) | Gobbur 2014 | Unclear | Unclear | Low | Unclear | Unclear | High | high |
| (15) | Goswami 2013 | Low | Low | Low | Unclear | Unclear | Low | some concern |
| (16) | Gungorduk 2010 | Low | Low | Low | Low | Low | Low | low |
| (17) | Gungorduk 2013 | Low | Low | Low | Low | Unclear | Low | some concern |
| (18) | Halifa 2021 | Low | Low | Low | Low | Low | Low | low |
| (19) | Hassan 2020 | Low | Low | Low | Low | Unclear | Low | some concern |
| (20) | Hasan 2021 | Low | Low | Low | Low | Low | Low | Low |
| (21) | Hemapriya 2020 | Unclear | Low | Low | Unclear | Unclear | Low | high |
| (22) | Ibrahim 2019 | Low | Low | Low | Low | Unclear | Low | some concern |
| (23) | Ifunanya 2019 | Low | Low | Low | Low | Unclear | Low | some concern |
| (24) | Igboke 2022 | Low | Low | Low | High | Low | Low | high |
| (25) | Ismail 2017 | Low | Low | Low | Low | Low | Low | low |
| (26) | Jafarbegloo 2017 | Low | Low | Low | Unclear | Low | Unclear | some concern |
| (27) | Kafayat 2018 | Low | Unclear | Unclear | Unclear | Unclear | High | high |
| (28) | Kamel 2018 | Low | Low | Low | High | Unclear | Low | high |
| (29) | Kashanian 2021 | Low | Low | Low | Low | Low | Low | low |
| (30) | Lakshmi 2016 | Low | High | Low | Low | Unclear | Low | high |
| (31) | Maged 2015 | Low | Low | Low | High | Low | Low | high |
| (32) | Matloob 2021 | Unclear | Unclear | Unclear | Low | Unclear | High | high |
| (33) | Mbah 2021 | Low | Low | Low | Low | Unclear | Low | some concern |
| (34) | Milani 2019 | Low | Low | Low | Unclear | Unclear | Low | some concern |
| (35) | Mirghafourvand 2015 | Low | Low | Low | Low | Low | Low | low |
| (36) | Movafegh 2011 | Low | Low | Low | Low | Unclear | Low | some concern |
| (37) | Naeiji 2021 | Low | Low | Low | Low | Low | Low | low |
| (38) | Nargis 2018 | Low | Low | Low | Low | Unclear | Low | some concern |
| (39) | Obi 2019 | Low | Low | Low | Low | Low | Low | low |
| (40) | Oseni 2021 | Low | Low | Low | Low | Unclear | Low | some concern |
| (41) | Ramani 2014 | Unclear | Unclear | Low | Unclear | Unclear | High | high |
| (42) | Ray 2016 | Low | High | Low | Low | Unclear | Low | high |
| (43) | Roy 2016 | Low | Low | Low | Low | Unclear | Low | some concern |
| (44) | Sekhavat 2009 | Low | Unclear | Low | Unclear | Unclear | Low | high |
| (45) | Sentilhes 2018 | Low | Low | Low | Low | Low | Low | low |
| (46) | Sentilhes 2021 | Low | Low | Low | Low | Low | Low | low |
| (47) | Senturk 2013 | Low | Low | Low | Low | Unclear | Low | some concern |
| (48) | Shah 2018 | Low | Unclear | Low | Unclear | Unclear | Low | high |
| (49) | Shahid 2013 | Low | Low | Low | Low | Unclear | Low | some concern |
| (50) | Sharma 2011 | Unclear | Unclear | Low | Low | Unclear | Unclear | high |
| (51) | Singh 2014 | Unclear | Unclear | Low | Low | Unclear | High | high |
| (52) | Soliman 2021 | Low | Low | Low | Low | Unclear | Low | some concern |
| (53) | Sujata 2016 | Unclear | Low | Low | Low | Low | Low | some concern |
| (54) | Sujita 2018 | Low | Low | Low | High | Unclear | Low | some concern |
| (55) | Tabatabaie 2021 | Unclear | Low | Low | Unclear | Unclear | Low | high |
| (56) | Taj 2014 | Unclear | Low | Unclear | Unclear | Unclear | Unclear | high |
| (57) | Torky 2020 | Low | Low | Low | Unclear | Unclear | Low | some concern |
| (58) | Xu 2012 | Low | Low | Low | Low | Unclear | Low | some concern |
| (59) | Yehia 2014 | Low | Low | Low | Low | Unclear | Low | some concern |

**Supplemental Data Legend:**

**Supplemental Digital Content, Figure 1***. Publication Bias*

**Supplemental Digital Content, Table 1***. Summary of the included studies*

**Supplemental Digital Content, Table 2***. Baseline characteristics of the included studies*

**Supplemental Digital Content, Table 3***. Assessment risk of bias*

References

1. Abdel-Aleem H, Alhusaini TK, Abdel-Aleem MA, Menoufy M, Gülmezoglu AM. Effectiveness of tranexamic acid on blood loss in patients undergoing elective cesarean section: Randomized clinical trial. Journal of Maternal-Fetal and Neonatal Medicine. 2013;26:1705-9.

2. Ahmed MR, Sayed Ahmed WA, Madny EH, Arafa AM, Said MM. Efficacy of tranexamic acid in decreasing blood loss in elective caesarean delivery. Journal of Maternal-Fetal and Neonatal Medicine. 2015;28:1014-8.

3. Ali MM, El-Bromboly WH, Elnagar WM, Hashem MFA. Prevention of postpartum hemorrhage after vaginal delivery using tranexamic acid. Egyptian Journal of Hospital Medicine. 2021;85:2937-40.

4. Nivedhana AP, Indu NR, Jalakandan B. Does prophylactic tranexamic acid reduce blood loss in Indian women following vaginal delivery? International Journal of Reproduction, Contraception, Obstetrics and Gynecology. 2021;10:497+.

5. G. B, MV A, Mittal S. Efficacy of prophylactic tranexamic acid in reducing blood loss during and after caesarean section. International Journal of Reproduction, Contraception, Obstetrics and Gynecology. 2016;5:2011-6.

6. Chandak AV, Gupta I. Efficacy of Tranexamic Acid in Decreasing Blood Loss during and after Cesarean Section: A Randomized Case Controlled Prospective Study. International Journal of Science and Research (IJSR) ISSN. 2015;6.

7. Diop A, Abbas D, Ngoc NTN, Martin R, Razafi A, Tuyet HTD, et al. A double-blind, randomized controlled trial to explore oral tranexamic acid as adjunct for the treatment for postpartum hemorrhage. Reproductive Health. 2020;17:1-7.

8. Ducloy-Bouthors AS, Jude B, Duhamel A, Broisin F, Huissoud C, Keita-Meyer H, et al. High-dose tranexamic acid reduces blood loss in postpartum haemorrhage. Critical Care. 2011;15:1-10.

9. Abd El-Gaber AE-N, Ahmed HH, Khodry MM, Abbas AM. Effect of tranexamic acid in prevention of postpartum hemorrhage in elective caesarean delivery: a randomized controlled study. International Journal of Reproduction, Contraception, Obstetrics and Gynecology. 2018;8:1.

10. El-Garhy ET, Mohamed AH, Elshahat A, Abu Elmagd I, Hamed MAA. Tranexamic Acid for Prevention of Postpartum Hemorrhage after Vaginal Delivery. The Egyptian Journal of Hospital Medicine. 2018;73(2):6157-64.

11. Fahmy NG, Eskandar FSL, Khalil WAMA, Sobhy MII, Amin AMAA. Assessment the role of tranexamic acid in prevention of postpartum hemorrhage. Ain-Shams Journal of Anesthesiology. 2021;13.

12. Farahat  MA. Role of intravenous tranexamic acid on cesarean blood loss: a prospective randomized study. Women's Health. 2019;8:226-30.

13. Gai MY, Wu LF, Su QF, Tatsumoto K. Clinical observation of blood loss reduced by tranexamic acid during and after caesarian section: A multi-center, randomized trial. European Journal of Obstetrics and Gynecology and Reproductive Biology. 2004;112:154-7.

14. Gobbur V, Shiragur S, Jhanwar U, Tehalia M. Efficacy of tranexamic acid in reducing blood loss during lower segment caesarean section. International Journal of Reproduction, Contraception, Obstetrics and Gynecology. 2014;3:414-7.

15. Goswami U, Sarangi S, Gupta S, Babbar S. Comparative evaluation of two doses of tranexamic acid used prophylactically in anemic parturients for lower segment cesarean section: A double-blind randomized case control prospective trial. Saudi Journal of Anaesthesia. 2013;7:427-31.

16. Gungorduk K, Yildirim G, Asicioǧlu O, Gungorduk OC, Sudolmus S, Ark C. Efficacy of intravenous tranexamic acid in reducing blood loss after elective cesarean section: A prospective, randomized, double-blind, placebo-controlled study. American Journal of Perinatology. 2011;28:233-9.

17. Gungorduk K, Asicioǧlu O, Yildirim G, Ark C, Tekirdaǧ A, Besimoglu B. Can intravenous injection of tranexamic acid be used in routine practice with active management of the third stage of labor in vaginal delivery? A randomized controlled study. American Journal of Perinatology. 2013;30:407-13.

18. Halifa I, Olusesan Oluwasola T, Fawole B, Oladokun A. Intravenous tranexamic acid for reducing blood loss during cesarean delivery: A double-blind, randomized-controlled trial. New Nigerian Journal of Clinical Research. 2021;10:40.

19. Hassan NE, Elghareeb NAM, Zaki FM. Efficacy of oxytocin infusion versus tranexamic acid infusion in controlling blood loss during elective lower segment caesarean section. Egyptian Journal of Hospital Medicine. 2020;81:1822-7.

20. Hasan CS, Alalaf SK, Khoshnaw SA. Tranexamic Acid Administration for the Prevention of Blood Loss After Vaginal Delivery in a High-Risk Pregnancy: A Double-blind Randomized Controlled Trial. 2021.

21. Hemapriya L, More G, Kumar A. Efficacy of Tranexamic Acid in Reducing Blood Loss in Lower Segment Cesearean Section: A Randomised Controlled Study. The Journal of Obstetrics and Gynecology of India. 2020;70(6):479-84.

22. Ibrahim TH. Efficacy of tranexamic acid in reducing blood loss, blood and blood products requirements in Cesarian sections for patients with placenta accreta. Ain-Shams Journal of Anesthesiology. 2019;11:2-7.

23. Ifunanya NJ, Chukwu IC, Nobert OC, Blessing O, Chibuzor UD-P, Uchenna OV. Tranexamic Acid versus Placebo for Prevention of Primary Postpartum Haemorrhage among High Risk Women Undergoing Caesarean Section in Abakaliki: A Randomized Controlled Trial. Open Journal of Obstetrics and Gynecology. 2019;09:914-22.

24. Igboke FN, Obi VO, Dimejesi BI, Lawani LO. Tranexamic acid for reducing blood loss following vaginal delivery: a double-blind randomized controlled trial. BMC Pregnancy Childbirth. 2022;22(1):178.

25. Ismail A, Abbas A, Shahat M, Ali K. Evaluation of Subendometrial and Intramyometrial Blood Flow after Intravenous Tranexamic Acid for Prevention of Postpartum Hemorrhage in Vaginal Delivery: A Randomized Controlled Study. Journal of Gynecological Research and Obstetrics. 2017;3:046-50.

26. Jafarbegloo E, Faridnyia F, Nejad ASMH. The Effect of Intravenous Tranexamic Acid on Hemoglobin and Hematocrit Levels After Cesarean Delivery: a Randomized Controlled Clinical Trial. 2021.

27. Kafayat H, Janjua M, Naheed I, Iqbal T. To assess the prophylactic role of tranexamic acid in reducing blood loss during and after two hours of caesarean section. Pakistan Journal of Medical and Health Sciences. 2018;12:1662-5.

28. Kamel HEH, Farhan AM, Abou Senna HF, Khedr MA, Albhairy AA. Role of Prophylactic Tranexamic Acid in Reducing Blood loss during Elective Caesarean section in Rural Area. The Egyptian Journal of Hospital Medicine. 2018;73:6886-96.

29. Kashanian M, Dadkhah F, Tabatabaei N, Sheikhansari N. Effects of tranexamic acid on the amount of bleeding following vaginal delivery and its adverse effects: a double-blind placebo controlled randomized clinical trial. Journal of Maternal-Fetal and Neonatal Medicine. 2021;0:1-5.

30. Dhivya Lakshmi SJ, Abraham R. Role of prophylactic tranexamic acid in reducing blood loss during elective caesarean section: A randomized controlled study. Journal of Clinical and Diagnostic Research. 2016;10:OC17-OC21.

31. Maged AM, Helal OM, Elsherbini MM, Eid MM, Elkomy RO, Dahab S, et al. A randomized placebo-controlled trial of preoperative tranexamic acid among women undergoing elective cesarean delivery. International Journal of Gynecology and Obstetrics. 2015;131:265-8.

32. Matloob M, Hyder Syed Z, Qasim R, Najeeb W. Comparison of Misoprostol With Tranexamic Acid in Preventing Post-Partum Hemorrhage. Pakistan Journal of Medical and Health Sciences. 2021;15(5):914-6.

33. Mbah K, Omietimi J, Oyeyemi N, Abasi I, Allagoa D, Oriji P, et al. The Efficacy of Prophylactic Tranexamic Acid in Reducing Perioperative Blood Loss During Caesarean Section: A Randomized, Double Blind Control Trial. Journal of Gynecology and Womens Health. 2021;21.

34. Milani F, Haryalchi K, Sharami SH, Atrkarroshan Z, Farzadi S. Prophylactic effect of tranexamic acid on hemorrhage during and after the cesarean section. International Journal of Women's Health and Reproduction Sciences. 2019;7:74-8.

35. Mirghafourvand M, Alizadeh Charandabi SM, Abasalizadeh F, Shirdel M. The effect of intravenous tranexamic acid on hemoglobin and hematocrit levels after vaginal delivery: A randomized controlled trial. Iranian Journal of Obstetrics, Gynecology and Infertility. 2013;16:1-8.

36. Movafegh A, Eslamian L, Dorabadi A. Effect of intravenous tranexamic acid administration on blood loss during and after cesarean delivery. International Journal of Gynecology and Obstetrics. 2011;115:224-6.

37. Naeiji Z, Delshadiyan N, Saleh S, Moridi A, Rahmati N, Fathi M. Prophylactic use of tranexamic acid for decreasing the blood loss in elective cesarean section: A placebo-controlled randomized clinical trial. Journal of Gynecology Obstetrics and Human Reproduction. 2021;50:101973.

38. Nargis N, Dewan F. Prophylactic use of tranexamic acid during caesarean section in preventing postpartum haemorrhage-a prospective randomised double blind placebo controlled study. Bangladesh Journal of Obstetrics and Gynecology. 2018;33:125-30.

39. Obi CN. Efficacy of intravenous tranexamic acid at reducing blood loss during elective caesarean section in Abakaliki: A double blind randomized placebo controlled trial. African Journal of Medical and Health Sciences. 2019;18:10-7.

40. Oseni RO, Zakari M, Adamou N, Umar UA. Effectiveness of preoperative tranexamic acid in reducing blood loss during caesarean section at Aminu Kano teaching hospital, Kano: A randomized controlled trial. Pan African Medical Journal. 2021;39.

41. Ramani B, Nayak L. Intravenous 1 gram tranexamic acid for prevention of blood loss and blood transfusion during caesarean section: a randomized case control study. International Journal of Reproduction, Contraception, Obstetrics and Gynecology. 2014;3:366-9.

42. Ray I, Bhattacharya R, Chakraborty S, Bagchi C, Mukhopadhyay S. Role of Intravenous Tranexamic Acid on Caesarean Blood Loss: A Prospective Randomised Study. Journal of Obstetrics and Gynecology of India. 2016;66:347-52.

43. Roy P, Sujatha MS, Bhandiwad A, Biswas B. Role of Tranexamic Acid in Reducing Blood Loss in Vaginal Delivery. Journal of Obstetrics and Gynecology of India. 2016;66:246-50.

44. Sekhavat L, Tabatabaii A, Dalili M, Farajkhoda T, Tafti AD. Efficacy of tranexamic acid in reducing blood loss after cesarean section. Journal of Maternal-Fetal and Neonatal Medicine. 2009;22:72-5.

45. Sentilhes L, Sénat MV, Le Lous M, Winer N, Rozenberg P, Kayem G, et al. Tranexamic Acid for the Prevention of Blood Loss after Cesarean Delivery. New England Journal of Medicine. 2021;384:1623-34.

46. Sentilhes L, Winer N, Azria E, Sénat M-V, Le Ray C, Vardon D, et al. Tranexamic Acid for the Prevention of Blood Loss after Vaginal Delivery. New England Journal of Medicine. 2018;379(8):731-42.

47. Sentürk MB, Cakmak Y, Yildiz G, Yildiz P. Tranexamic acid for cesarean section: A double-blind, placebo-controlled, randomized clinical trial. Archives of Gynecology and Obstetrics. 2013;287:641-5.

48. Shah P, Agrawal A, Chhetri S, Rijal P, Bhatta NK. Tranexamic acid in prevention of postpartum hemorrhage in elective cesarean section. International Journal of Reproduction, Contraception, Obstetrics and Gynecology. 2019;8:372.

49. Shahid A, Khan A. Tranexamic acid in decreasing blood loss during and after caesarean section. J Coll Physicians Surg Pak. 2013;23(7):459-62.

50. Sharma R, Najam R, Misra MK. Efficacy of Tranexamic Acid in Decreasing Blood Loss During and After Cesarean Section. Biomedical and Pharmacology Journal. 2011;4(1):231-5.

51. Singh T, Burute SB, Deshpande HG, Jethani S, Ratwani K. Efficacy of Tranexamic Acid in Decreasing Blood Loss During and After Caesarean Section: a Randomized Case Control Prospective Study. Journal of Evolution of Medical and Dental Sciences. 2014;3:2780-8.

52. Soliman AA, Mahmoud SA, Dawood RM, Fayed AA, Fathey AA. Prophylactic use of tranexamic acid in reducing blood loss during elective cesarean section. Egyptian Journal of Hospital Medicine. 2021;82:6-10.

53. Sujata N, Tobin R, Kaur R, Aneja A, Khanna M, Hanjoora VM. Randomized controlled trial of tranexamic acid among parturients at increased risk for postpartum hemorrhage undergoing cesarean delivery. International Journal of Gynecology and Obstetrics. 2016;133:312-5.

54. Sujita A, Songthamwat S, Songthamwat M. Effectiveness of tranexamic acid for reducing postpartum blood loss in the first two hours after vaginal delivery: A randomised controlled trial. Journal of Clinical and Diagnostic Research. 2018;12:QC01-QC4.

55. Tabatabaie SS, Alavi A, Bazaz M. Comparison of the effect of tranexamic acid and misoprostol on blood loss during and after cesarean section: A randomized clinical trial. Razavi International Journal of Medicine. 2021;9:e811.

56. Efficacy of Tranexamic acid in reducing blood loss during and after Cesarean section, (2014).

57. Torky H, El-Desouky ES, Abo-Elmagd I, Mohamed A, Abdalhamid A, El-Shahat A, et al. Pre-operative tranexemic acid vs. Etamsylate in reducing blood loss during elective cesarean section: Randomized controlled trial. Journal of Perinatal Medicine. 2021;49:353-6.

58. Xu J, Gao W, Ju Y. Tranexamic acid for the prevention of postpartum hemorrhage after cesarean section: A double-blind randomization trial. Archives of Gynecology and Obstetrics. 2013;287:463-8.

59. Yehia AH, Koleib MH, Abdelazim IA, Atik A. Tranexamic acid reduces blood loss during and after cesarean section: A double blinded, randomized, controlled trial. Asian Pacific Journal of Reproduction. 2014;3:53-6.
