# Supplementary figures and images for: Efficacy and safety of tranexamic acid in prevention of postpartum hemorrhage: a systematic review and meta-analysis of 18,649 patients
Source: BMC Pregnancy Childbirth. 2023 Nov 24;23:817. doi: 10.1186/s12884-023-06100-8 (PMC10668444; doi:10.1186/s12884-023-06100-8)

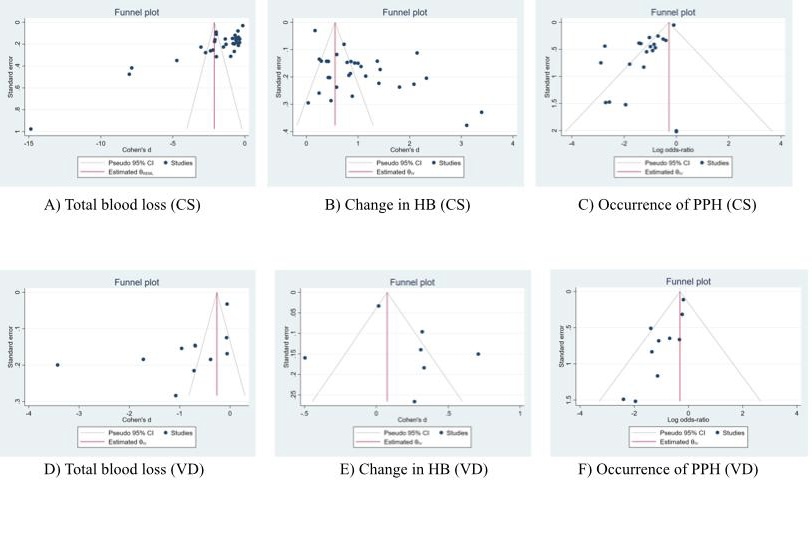

Supplement: Supplementary file 4 — Additional file 4: Supplementary Figure 1: Visual inspection of funnel plots in terms of total blood loss, change in hemoglobin, and occurrence rate of PPH. [file 12884_2023_6100_MOESM4_ESM.jpg]
